# Diaryl- and triaryl-pyrrole derivatives: inhibitors of the MDM2–p53 and MDMX–p53 protein–protein interactions†
†Electronic supplementary information (ESI) available: Experimental details for compound synthesis, analytical data for all compounds and intermediates. Details for the biological evaluation. Further details for the modeling. Table of combustion analysis data. See DOI: 10.1039/c3md00161jClick here for additional data file.



**DOI:** 10.1039/c3md00161j

**Published:** 2013-07-29

**Authors:** Tim J. Blackburn, Shafiq Ahmed, Christopher R. Coxon, Junfeng Liu, Xiaohong Lu, Bernard T. Golding, Roger J. Griffin, Claire Hutton, David R. Newell, Stephen Ojo, Anna F. Watson, Andrey Zaytzev, Yan Zhao, John Lunec, Ian R. Hardcastle

**Affiliations:** a Newcastle Cancer Centre , Northern Institute for Cancer Research and School of Chemistry , Newcastle University , Bedson Building , Newcastle , NE1 7RU , UK . Email: Ian.Hardcastle@ncl.ac.uk ; Fax: +44 (0)191 8591 ; Tel: +44 (0)191 222 6645; b Newcastle Cancer Centre , Northern Institute for Cancer Research , Newcastle University , Paul O'Gorman Building, Medical School, Framlington Place , Newcastle , NE2 4HH , UK . Email: John.Lunec@ncl.ac.uk ; Fax: +44 (0)191 4301 ; Tel: +44 (0)191 246 4420

## Abstract

Screening identified 2-(3-((4,6-dioxo-2-thioxotetrahydropyrimidin-5(2*H*)-ylidene)methyl)-2,5-dimethyl-1*H*-pyrrol-1-yl)-4,5,6,7-tetrahydrobenzo[*b*]thiophene-3-carbonitrile as an MDM2–p53 inhibitor (IC_50_ = 12.3 μM). MDM2–p53 and MDMX–p53 activity was seen for 5-((1-(4-chlorophenyl)-2,5-diphenyl-1*H*-pyrrol-3-yl)methylene)-2-thioxodihydropyrimidine-4,6(1*H*,5*H*)-dione (MDM2 IC_50_ = 0.11 μM; MDMX IC_50_ = 4.2 μM) and 5-((1-(4-nitrophenyl)-2,5-diphenyl-1*H*-pyrrol-3-yl)methylene)pyrimidine-2,4,6(1*H*,3*H*,5*H*)-trione (MDM2 IC_50_ = 0.15 μM; MDMX IC_50_ = 4.2 μM), and cellular activity consistent with p53 activation in MDM2 amplified cells. Further SAR studies demonstrated the requirement for the triarylpyrrole moiety for MDMX–p53 activity but not for MDM2–p53 inhibition.

## Abstract 2

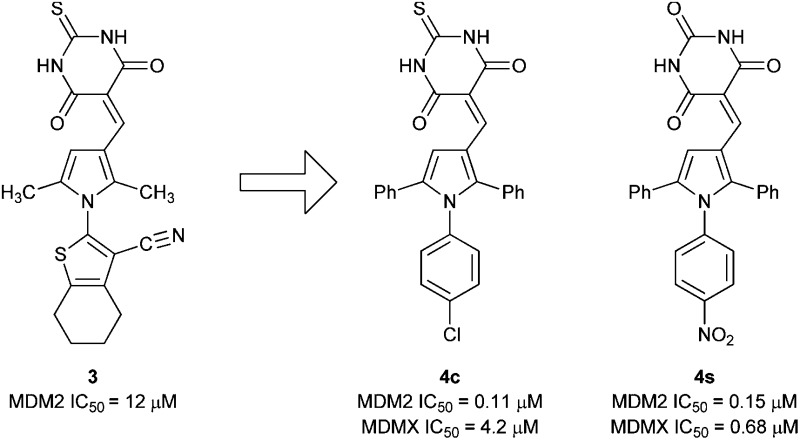
Triarylpyrroles *e.g.* **4c** and **4s** inhibit the MDM2–p53 and MDMX–p53 protein–protein interactions.

## Introduction

The tumor suppressor protein p53 functions as a molecular sensor in diverse signalling pathways resulting from cellular stresses, such as DNA damage, oncogene activation and possibly hypoxia.^1^ Abrogation of the function of the tumor suppressor p53 is a key feature of the molecular pathology in many cancers. Mutation of the *TP53* gene occurs in approximately 50% of common adult sporadic cancers, resulting in inactive protein.^2,3^ Alternatively, p53 may be silenced by the overexpression of the regulatory proteins MDM2 or MDMX (MDM4).^4–6^ MDM2 amplification has been reported to occur in approximately 11% of all tumors and the paralogue MDMX has been reported to be amplified in brain (11%), breast (5–40%), and soft tissue tumors (17%). Overexpression of MDMX has also been observed in a wider range of tumor types, including uterus (15%), testes (27%), melanoma (65%), stomach/small intestine (43%), and lung (18%).^6,7^

The MDM2 and MDMX proteins regulate the activity of p53 with different and non-redundant mechanisms.^8^ In addition to the *MDM2* gene being a target for p53-dependent transcription, MDM2 regulates p53 in an autoregulatory negative feedback loop by binding to the p53 transactivation domain, and acting as an E3-ligase for polyubiquitination of p53 to promote p53 degradation by the ubiquitin-mediated proteosomal pathway.^9–12^ MDMX also inhibits p53 transcriptional activity, but does not act as an E3 ligase independently of MDM2, and its expression is not p53 dependent.^13^ Furthermore, MDMX–MDM2 heterodimers have enhanced E3 ligase activity over MDM2 alone and may be an important mechanism of p53 regulation.

The MDM2–p53 binding interaction is amenable to small-molecule inhibition, as it consists of a relatively deep binding groove on the surface of the MDM2 protein into which an amphipathic helix of p53 binds.^14^ A number of potent MDM2–p53 inhibitors have been reported based on diverse chemotypes,^15^ such as the *cis*-imidazoline RG-7112 (IC_50_ = 12 nM),^16^ spirooxindoles, *e.g.* MI-888 (IC_50_ = 6.8 nM),^17^ and the substituted piperidone AM-8553 (IC_50_ = 2.2 nM),^18^ and have demonstrated cellular activity consistent with inhibition of MDM2–p53 binding and *in vivo* antitumor activity. However, these series lack significant potency against MDMX,^19^ and overexpression of MDMX offers a possible mechanism of resistance to such MDM2–p53 inhibitors. For this reason compounds able to inhibit both interactions have great significance.^20^

To date, there have been few reports of small-molecule MDMX inhibitors. The 5-oxo-pyrazolylidene SJ-172552 was identified in an MDMX high-throughput fluorescence polarisation assay and showed selective MDMX inhibition, through a complex, irreversible mechanism.^21,22^ The 3-imidazolyl indole (**1a**) is a mixed MDM2–, MDMX–p53 inhibitor (MDM2 IC_50_ = 0.19 μM; MDMX IC_50_ = 20 μM), and has provided the first X-ray crystal structure of MDMX bound to a small-molecule ligand.^19^ A series of MDM2–p53 inhibitory pyrrolidone derivatives, *e.g.* (**2a**, MDM2 = 0.26 μM, MDMX = 2.7 μM; and **2b**, MDM2 = 1.3 μM, MDMX = 2.1 μM), also show modest MDMX activity in addition to MDM2 inhibition.^23^ The indolyl hydantoins, *e.g.* RO-5963 (MDM2 = 17 nM, MDMX = 25 nM), are the most potent MDM2–p53 and MDMX–p53 inhibitors reported to date.^24^ 
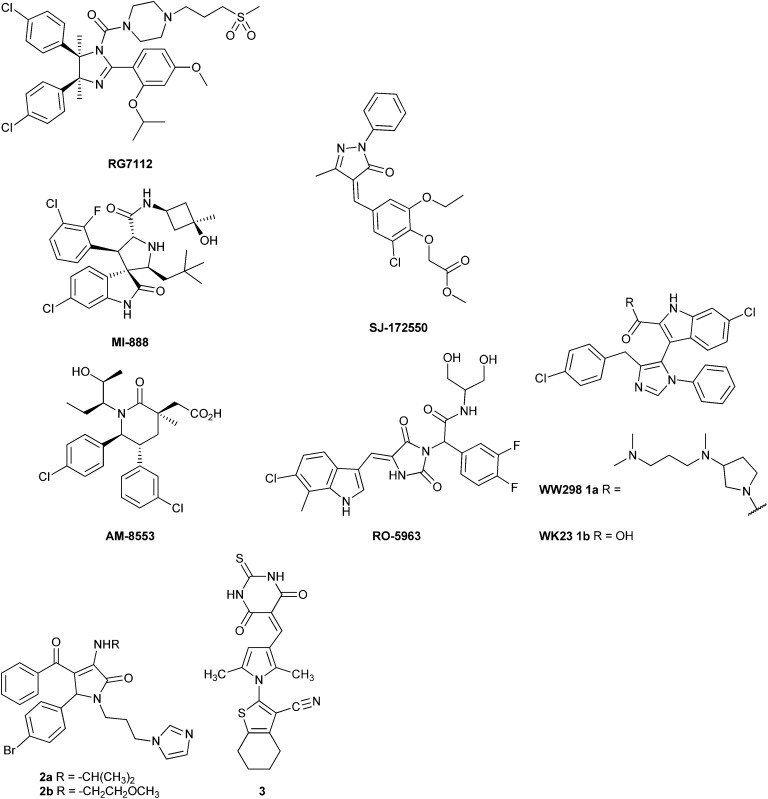


In this paper, we describe the discovery, structure–activity relationships (SARs) and cellular activity of triarylpyrrole compounds with promising inhibitory activity against both MDM2–p53 and MDMX–p53. Comparison of molecular models of the triarylpyrroles with a small series of the related diarylpyrrole MDM2–p53 inhibitors demonstrates key structural requirements for mixed MDM2 and MDMX inhibition in this series.

### Identification of MDM2–p53 inhibitors by screening

A pilot set of 800 structurally diverse compounds, obtained from the Cancer Research UK screening collection was studied in an MDM2–p53 ELISA protein–protein binding assay, at 5 and 20 μM concentrations.^25^ Follow up IC_50_ determinations on active compounds revealed pyrrole **3** as a hit, with an IC_50_ of 12.3 ± 1.5 μM against MDM2–p53, which also demonstrated dose-dependent cellular activity by Western blotting for MDM2 and p53 induction. A series of 96 related analogues was purchased, based on similarity searching and visual inspection, and screened for MDM2 activity. Twelve compounds (**4a–l**) showed promising MDM2–p53 inhibitory activity with IC_50_ values in the 0.12–8.4 μM range (Table 1). Pyrroles bearing *N* ^2^-aryl groups with chloro-, bromo-, or ethoxylcarbonyl 4-substituents showed the greatest potency. Pyrroles with 2,5-diphenyl substitution (**4c**, **4d**) were 5–10 fold more potent than the comparable 2,5-dimethyl analogues (**4a**, **4b**). Substitution on the thiobarbituric acid moiety had a modest negative effect on potency.

**Table 1:** MDM2 structure–activity relationships for purchased pyrroles **4a–l**

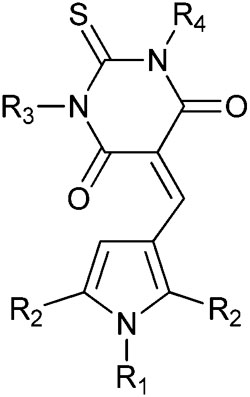					
Compound	R^1^	R^2^	R^3^	R^4^	MDM2 IC_50_ (nM)
**4a**	4-ClPh	Me	H	H	720 ± 100
**4b**	4-ClPh	Me	H	Ph	3300 ± 700
**4c**	4-ClPh	Ph	H	H	120 ± 20
**4d**	4-ClPh	Ph	H	Ph	230 ± 44
**4e**	4-ClPh	Ph	H	3-ClPh	163 ± 17
**4f**	4-ClPh	Ph	3,4-diMePh	Ph	256 ± 39
**4g**	4-ClPh	Ph	Me	Me	Insol.
**4h**	4-BrPh	Me	Ph	Ph	199 ± 16
**4i**	4-MePh	Me	H	Ph	8400 ± 900
**4j**	4-MePh	Me	H	H	4700 ± 200
**4k**	4-EtO_2_CPh	Ph	H	H	700 ± 20

### Synthesis and structure–activity relationships

**Scheme 1:**
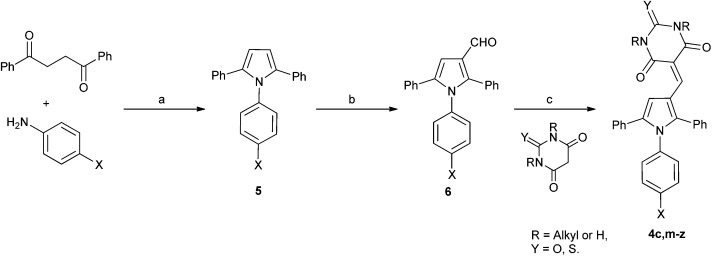
Synthesis of substituted triarylpyrroles **4a–z**. *Reagents and conditions*: (a) TFA, TFE, MW, 150 °C, 20 min; (b) POCl_3_ DMF, 0–70 °C, MW, 1 h; (c) EtOH, rt, 12 h, or AcOH, 120 °C, 2 h.

In order to validate and further explore the SAR, a selection of 2,5-symmetrically substituted pyrroles **5** was prepared from 1,2-dibenzoylethane and the appropriate 4-substituted aniline, using trifluoroethanol (TFE) as solvent and trifluoroacetic acid (TFA) as catalyst under microwave heating (Scheme 1).^26–29^ The use of TFE-TFA with microwave heating is especially advantageous over conventional procedures^30^ as it ensures clean, homogeneous reactions that proceed in high yields. Formylation of the pyrroles under Vilsmeier–Haack conditions proceeded smoothly with microwave heating to give 3-formylpyrroles **6** in good yields. Condensation of aldehydes **6** with the required barbituric or thiobarbituric acid was carried out in ethanol at room temperature or in acetic acid at 120 °C to give the pyrrole barbiturates (**4a–w**). The use of mono-*N*-methyl barbituric or thiobarbituric acid gave **4x–z** as inseparable mixtures of regioisomers.

MDM2 inhibitory SARs for a series of analogues of **4c** were determined, and selected compounds were assayed for MDMX–p53 inhibitory activity (Table 2). Unexpectedly, pyrrole **4c** was shown to be a low micromolar inhibitor of the MDMX–p53 interaction. Comparision of the MDM2 inhibitory activity of matched pairs of thiobarbituric acid and barbituric acid derivatives shows a 3–20 fold reduction in potency for the oxo-derivatives (*e.g.* **4m** and **4c**, **4q** and **4r**, **4v** and **4w**, **4y** and **4z**), with the exception of the 4-nitro derivatives **4s** and **4t** that were equipotent. The 4-*N*-aryl substitutents had a profound influence on potency for MDM2–p53. Thus, potency was conferred by chloro- or bromo-substituents (**4c**, **4m**, and **4n**) or electron-withdrawing groups *e.g.* nitro or cyano (**4q–t**). In contrast, larger or electron-donating groups gave poor MDM2 inhibition, *e.g.* OCH_3_, *t*-Bu (**4o** and **4p**). The 4-aryl substituent also significantly influenced activity against MDMX, with 4-nitro- (**4s** and **4t**), 4-cyano- (**4q** and **4r**), or 4-chloro- (**4c**) *N*-phenyl substituents conferring the greatest inhibitory potency. The *N*,*N*-diethylbarbituric acid or thiobarbituric acid derivatives (**4u,v**) were 3–4 fold less potent against MDM2 compared with their unsubstituted analogues, whereas the mono-*N*-methyl analogues (**4x,y**) were equipotent with their parents. Similarly, *N*-alkyl substitution on the barbituric acid or thiobarbituric acid moiety (**4u–z**) resulted in a significant loss of MDMX–p53 activity. In all cases reduced solubility was observed for the *N*-alkyl derivatives.

**Table 2:** MDM2 and MDMX structure–activity relationships for triarylpyrroles **4c** and **4m–z**

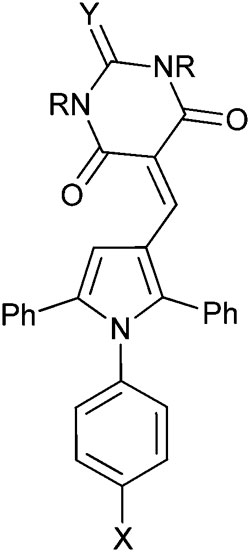						
Compound	R^1^	R^2^	X	Y	MDM2 IC_50_ (μM)^*a*^	MDMX IC_50_ (μM)^*a*^
**4c**	H	H	Cl	S	0.11 ± 0.03	4.2 ± 1.2
**4m**	H	H	Cl	O	0.30 ± 0.03	nd
**4n**	H	H	Br	O	0.18 ± 0.07	nd
**4o**	H	H	OMe	O	1.9 ± 0.3	13 ± 7
**4p**	H	H	*t*-Bu	O	1.9 ± 0.3	nd
**4q**	H	H	CN	O	4.7 ± 1.9	7.0 ± 3.0
**4r**	H	H	CN	S	0.20 ± 0.07^*d*^	0.90 ± 0.42
**4s**	H	H	NO_2_	O	0.15 ± 0.06	0.68 ± 0.18
**4t**	H	H	NO_2_	S	0.17 ± 0.09^*d*^	0.63 ± 0.12
**4u**	Et	Et	Cl	S	0.30 ± 0.12^*c*^	nd
**4v**	Et	Et	Br	O	0.89 ± 0.04	74%^*b*^
**4w**	Et	Et	Br	S	0.26 ± 0.05	nd
**4x**	Me	H	Cl	S	0.11 ± 0.02^*d*^	28 ± 23
**4y**	Me	H	Br	O	0.34 ± 0.08	35 ± 20
**4z**	Me	H	Br	S	0.073 ± 0.002	nd

^*a*^ *n* = 3, from resynthesised material.

^*b*^% inhibition at 50 μM.

^*c*^ *n* = 6.

^*d*^ *n* = 4; nd = not determined.

**Scheme 2:**
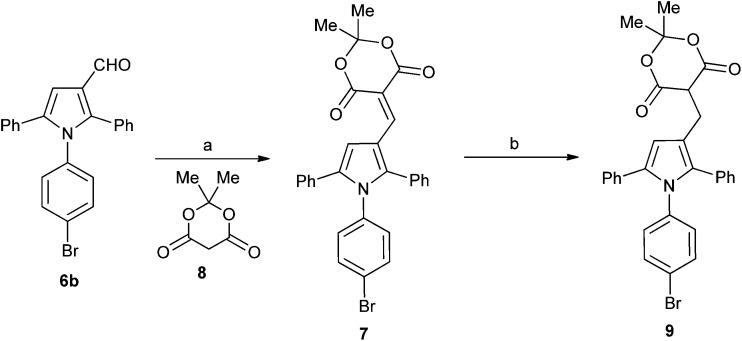
Synthesis of substituted triarylpyrroles **7** and **7**. *Reagents and conditions*: (a) toluene, piperidine, acetic acid, Δ; (b) NaBH_4_, EtOH.

**Scheme 3:**
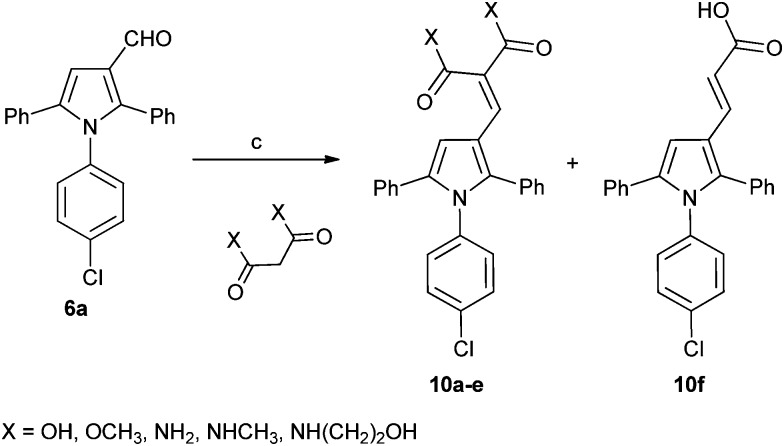
Synthesis of substituted triarylpyrroles **10a–f**. *Reagents and conditions*: (a) toluene, piperidine, acetic acid, Δ.

A series of derivatives with alternative substituents to the barbituric acid was prepared to explore the SAR for this moiety for both MDM2 and MDMX inhibition. Compound **7** was prepared by heating aldehyde **5b** with Meldrum's acid **8** in toluene with piperidine acetate as catalyst (Scheme 2). Reduction of **7** to **9** was achieved with sodium borohydride in ethanol. The malonic acid derivatives **10a–e** were prepared by condensation of the required malonic acid, ester or amide with aldehyde **5a** (Scheme 3). The reaction with malonic acid gave the decarboxylated product **10f** in addition to **10b**.

The Meldrum's acid derivative **7** was >100-fold less potent than **8c** against MDM2–p53, whereas the reduced derivative **9** suffered a less significant 20-fold reduction in potency (Table 3). The acyclic derivatives **10a–f** all lacked significant MDMX–p53 activity, and showed reduced MDM2–p53 potency compared with **4c**. The dimethyl malonate derivative **10a** was essentially inactive, whereas the malonic acid derivative **10b** showed modest activity against MDM2–p53. The malonamide derivative **10c** showed similar activity to **10b**, whereas the *N*-alkyl malonamide derivatives **10d** and **10e** showed a 3- and 6-fold loss of potency against MDM2–p53. The monocarboxylic acid analogue **10f** was only weakly active against MDM2–p53 and inactive against MDMX–p53. These results suggest that binding to MDM2 requires at least one H-bond donor in the 4-substituent.

**Table 3:** MDM2 and MDMX structure–activity relationships for triarylpyrroles **7**, **9**, and **10a–f**

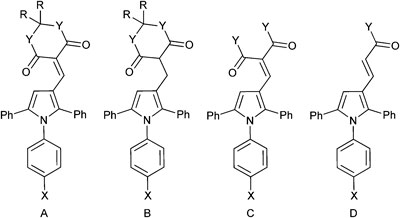						
Compound	Structure	R	X	Y	MDM2 IC_50_ (μM)^*a*^	MDMX IC_50_ (μM)^*a*^
**7**	A	Me	Br	O	17 ± 4^*b*^	nd
**9**	B	Me	Br	O	2.9 ± 0.7^*b*^	nd
**10a**	C	—	Cl	OMe	>50	>50
**10b**	C	—	Cl	OH	2.9 ± 0.2	>50
**10c**	C	—	Cl	NH_2_	2.5 ± 0.2	30^*c*^
**10d**	C	—	Cl	NHMe	7.6 ± 0.4	30^*c*^
**10e**	C	—	Cl	NH(CH_2_)_2_OH	15 ± 3	42^*c*^
**10f**	D	—	Cl	OH	17 ± 3	>50

^*a*^ *n* = 3.

^*b*^ *n* = 5.

^*c*^ *n* = 1; nd = not determined.

**Scheme 4:**
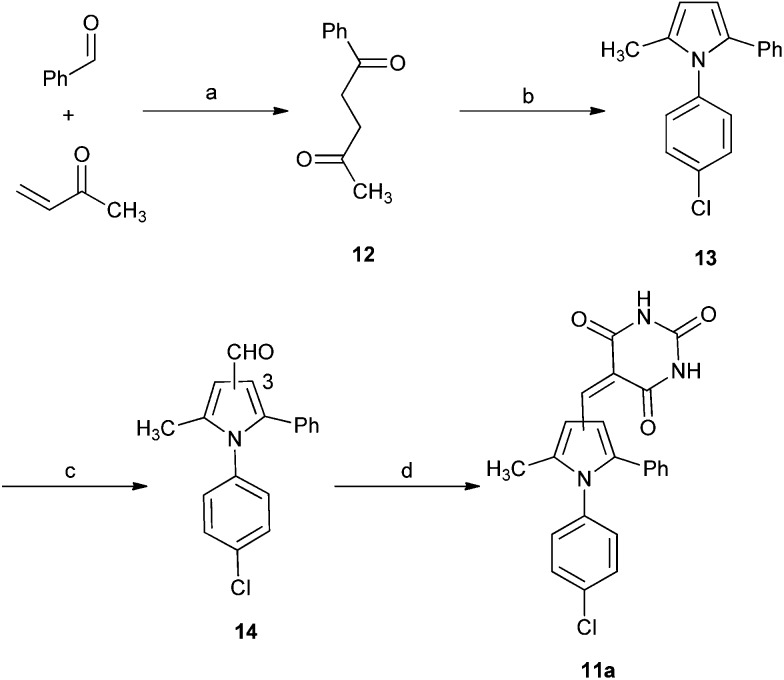
Synthesis of substituted diarylpyrrole **11a**. *Reagents and conditions*: (a) 3-benzyl-5-(2-hydroxyethyl)-4-methylthiazolium chloride, NEt_3_, RT; (b) TFA, TFE, MW, 150 °C, 20 min; (c) POCl_3_, DMF, 0–70 °C MW, 1 h; (d) barbituric acid, AcOH, 120 °C.

**Scheme 5:**
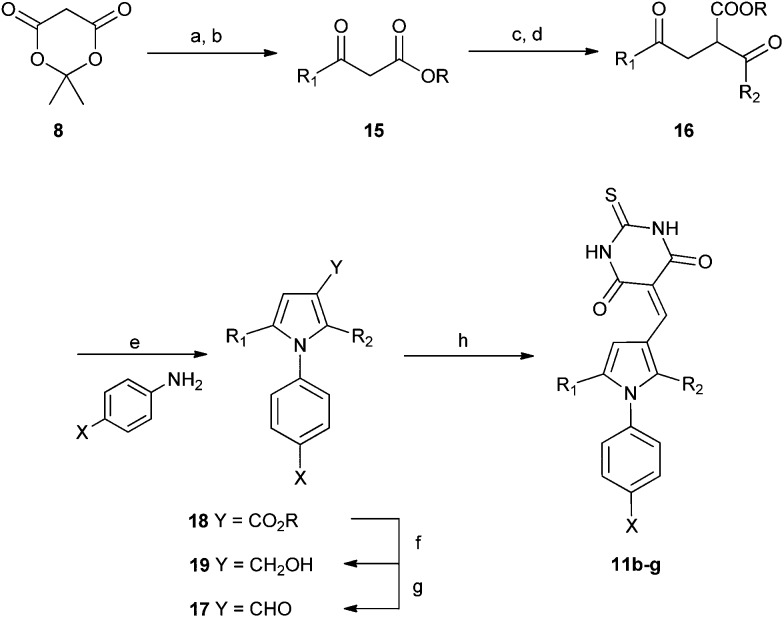
Synthesis of substituted diarylpyrroles **11b–g**. *Reagents and conditions*: (a) pyridine, R^1^COCl, DCM, 0 °C; (b) EtOH, toluene, reflux; (c) CH_2_I_2_, Et_2_Zn, R^2^CHO DCM 0 °C; (d) PCC, DCM, R.T.; (e) TFA, TFE, MW, 150 °C or PTSA, toluene, Dean–Stark; (f) DIBAL-H, DCM, –78 °C; (g) TPAP, NMO, 4 Å MS, DCM, rt; (h) thiobarbituric acid, AcOH, 120 °C-rt.

A series of 2-alkyl-1,5-diarylpyrroles **11** was designed to probe the SAR about the pyrrole for MDM2 and MDMX inhibition (Table 4). Their synthesis required the preparation of 1,4-diketones **12** *via* a Stetter reaction followed by cyclisation with the appropriate aniline (Scheme 4).^31^ Formylation of pyrrole **13** gave an inseparable mixture of isomers (**14**) that was reacted with barbituric acid affording a mixture of 3- and 4-isomers **11** that were only separable by HPLC (*e.g.* X = Cl). The limited practicality of this route prompted the search for a method capable of yielding either regioisomer, as required. Thus, β-ketoesters **15**, prepared from Meldrum's acid **12**, were subjected to a tandem homologation/addition sequence mediated by the Furukawa reagent,^32^ with oxidation of the intermediate providing the α-ester 1,4-diketones **16** (Scheme 5).^27^ Regiospecific synthesis of 3-, and 4-substituted pyrroles was achieved by variation of the R-groups on the β-ketoester and the aldehyde, providing both regioisomeric α-ester 1,4-diketones **16** that were combined with the required aniline. This is a versatile and efficient approach to a variety of 1,2,3-trisubstituted pyrroles. The aldehydes **17** were prepared from the esters **18** by DIBAL-H reduction to the corresponding alcohols **19** followed by oxidation.^33^ Condensation with thiobarbituric acid gave the desired non-symmetrically substituted pyrroles **11b–g**.

**Table 4:** MDM2 and MDMX structure–activity relationships for diarylpyrroles **11a–g**

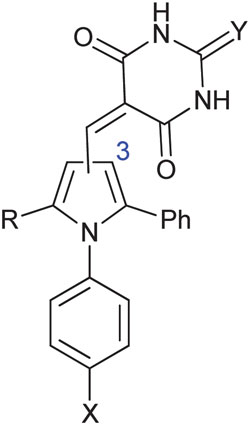						
Compound	Isomer	R^1^	X	Y	MDM2 IC_50_ ^*a*^ (μM)	MDMX IC_50_ ^*b*^ (μM)
**11a**	Mixture	Me	Cl	O	>1	nd
**11b**	3	*t*-Bu	Br	S	0.76 ± 0.27^*c*^	963
**11c**	3	*t*-Bu	Cl	S	1.1 ± 0.7^*c*^	1684
**11d**	4	CyPr	Cl	S	1.6 ± 1.7	3486
**11e**	4	CyPr	Br	S	1.6 ± 1.6	3428
**11f**	3	CyPr	Cl	S	2.1 ± 2.7	4916
**11g**	3	CyPr	Br	S	2.2 ± 2.7	5322

^*a*^ *n* = 3.

^*b*^ *n* = 1.

^*c*^ *n* = 4; nd = not determined.

Substitution of the 2-phenyl residue with a methyl group resulted in a greater than 3-fold loss of MDM2 inhibitory potency for the mixture of regioisomers **11a** (Table 4). Replacement of the 5-phenyl with a *tert*-butyl group (**11b,c**) gave a small loss of MDM2 inhibitory potency, independent of the position of the thiobarbituric acid residue, but resulted in *a* >200 fold loss of potency for MDMX. Similarly, the 2- or 5-cyclopropyl derivatives retained modest MDM2 inhibitory activity, independent of the position of the thiobarbituric acid residue, but were inactive against MDMX.

### MDM2 and MDMX modeling studies

Previously, we have demonstrated that docking ligands into the MDM2 binding pocket can yield multiple low energy solutions.^34^ For this reason, we opted to generate representative binding modes for the pyrroles from the X-ray co-crystal structures of MDM2 and MDMX with structurally similar ligands, *i.e.* imidazole derivatives (**1a,b**), by superposition and replacement of the ligand.^19^ Pyrrole (**4c**) with mixed MDM2 and MDMX potency, and two MDM2 selective pyrroles (**11c** and **11d**) were aligned in the ligands **1a** in MDM2 (pdb: ; 3LBK) and **1b** in MDMX (pdb: ; 3LBJ), using the ligand builder function and the CCP4 ‘cprodrg’ plugin within COOT.^35,36^ The high degree of structural overlap between the original ligand and the modeled compound in these models gives confidence that the binding modes are reasonable.

**Fig. 1:**
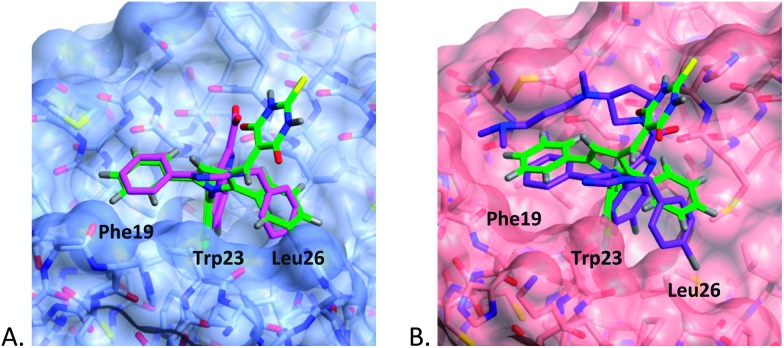
Modeled binding mode of **4c** (green) overlayed with: (A) **1b** (magenta) in MDM2 (light blue); (B) **1a** (purple) in MDMX (pink), binding pockets for p53 residues are indicated.

The binding modes for **4c** in both MDM2 and MDMX (Fig. 1) show good overlap with two of the aryl substituents of the imidazole series (**1a,b**). In particular, the *N*-4-chlorophenyl ring occupies the pocket normally filled by Trp23 of p53 for both MDM2 and MDMX,^14^ overlaying the chloroindole ring of **1a** or **1b**. The 5-phenyl ring of the pyrrole occupies the Phe19 pocket with good overlap with the 1-phenyl ring of **1a** or **1b**. The remaining phenyl ring is accommodated by the Leu26 pocket with a less well defined overlap and different vector compared with the original ligands. The thiobarbituric acid group projects away from the protein surface into the space occupied by the carboxylic acid residue of **1a** and the amide group of **1b**, suggesting that these groups may act, in part, as a hydrophilic cap.^37^

The MDM2 binding mode model is consistent with the observed SARs, as the Trp23 pocket of MDM2 shows a strong preference for haloaromatic groups as seen in the X-ray structures of high-affinity ligands. The preference for haloaromatic groups in the MDMX Trp23 binding-pocket is not as well established as for MDM2 due to the smaller number of deposited structures; however, the SARs in this series suggest that the pocket is similar to that in MDM2.

The role of the barbituric acid or thiobarbituric acid group is less well explained by the models. The positioning of the groups in the models is consistent with that seen for the amide of **1a** bound to MDMX and the acid group of **1b** bound to MDM2, and raises the possibility that water mediated H-bonding to the protein backbone may be important for affinity.

**Fig. 2:**
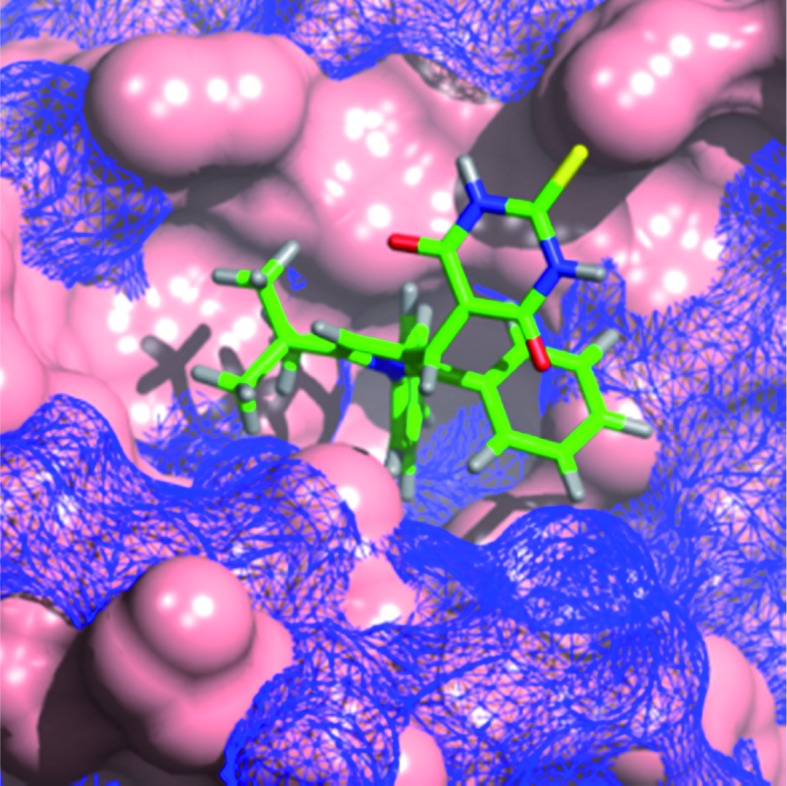
Modeled binding mode of 1,2-diarylpyrrole **11c** (green) overlayed with MDM2 (blue mesh) and MDMX (solid pink).

The modelling of **11c** into MDM2 and MDMX shows the *N*-4-chlorophenyl group occupying the Trp23 pocket as seen for **4c** (Fig. 2). The *t*-butyl residue is positioned into the Phe19 binding pocket, which is occupied by a number of alkyl substituents in recent MDM2 X-ray structures, *e.g.* the MI-series (3LBL)^19^ and the AM-8553 series (4ERE).^18^ The MDMX structure also places the *t*-butyl group into the Phe19 pocket, but the 5-phenyl ring no longer makes a good interaction with Leu26 pocket which appears to be broader and shallower than for MDM2. This observation may explain the dramatic loss in MDMX potency for this series, compared with the retention of MDM2 potency. Interestingly, the model of **11d** (ESI†), demonstrates the same arrangement of substituents, regardless of the positioning of the thiobarbituric acid moiety on the pyrrole ring.

### Cellular activity of pyrrole inhibitors

Growth inhibitory activity was determined for selected pyrroles in a panel of cell lines with defined MDM2, MDMX and p53 status (Table 5). The SJSA-1 osteosarcoma line has amplified *MDM2* and wild-type p53, and is paired with the SN40R2 line (MDM2 amplified, p53 mutant), an SJSA-1 derived line that is resistant to Nutlin-3a due to p53 mutation. For comparison, the MRK-NU-1 breast cancer cell line has amplified *MDMX* and wild-type p53. Compounds showed growth inhibitory activity in the 2–10 μM range without a strong correlation to either MDM2 or MDMX inhibitory activity. Disappointingly, the compounds were equally growth inhibitory in all cell lines regardless of their potency *vs.* MDM2 or MDMX, and the MDM2 and MDMX status of the cell line. Importantly, the p53 mutant SN40R2 line was equally sensitive to the pyrroles **4c**, **4r**, **4t** and **4x**, in contrast to the 2–100 fold difference in activity reported for potent MDM2 inhibitors.

**Table 5:** Growth inhibitory activity of pyrroles **4c**, **4r**, **4t**, **4x** in a panel of cell lines with defined MDM2, MDMX and p53 status^*a*^

Compound	GI_50_ (μM)		
	SJSA-1	SN40R2^*b*^	MRK-NU-1
**4c** ^*c*^	2.3 ± 0.2	2.8 ± 0.6	2.3 ± 0.3
**4r** ^*c*^	5.1 ± 0.9	5.9 ± 0.3	6.1 ± 0.7
**4t** ^*c*^	4.7 ± 1.0	5.2 ± 0.4	4.9 ± 0.6
**4x** ^*c*^	5.5 ± 0.6	6.3 ± 0.4	7.0 ± 1.0

^*a*^ *n* = 3.

^*b*^p53 null.

^*c*^Resynthesised material.

**Fig. 3:**
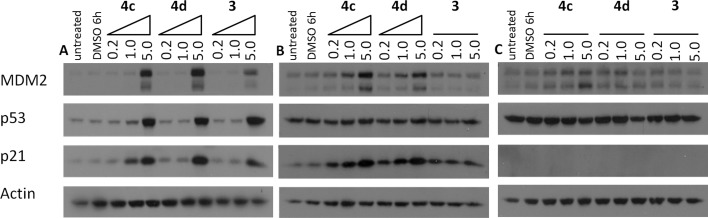
Cellular activity of **3**, **4c** and **4d** for p53 pathway activation detected by western blotting in cell lines treated with increasing concentrations (μM) for 4 h: (A) SJSA-1 cells; (B) A2780 cells; and (C) A2780CP70 cells.

Cell lines with defined MDM2 and p53 status were treated with increasing concentrations (0.1, 0.2 and 5 μM) of pyrroles **3**, **4c** and **4d** to investigate the transcriptional activation of p53 and the subsequent induction of p53-dependent proteins by Western blotting. In the SJSA-1 line (MDM2 amplified, p53wt) induction of MDM2, p53 and p21 was clearly visible at 5 μM for each compound (Fig. 3). In the A2780 line (p53wt) induction of MDM2 and p21 is observed for **4c** and **4d**, but not for **3**. Higher levels of p53 obscured any induction in this case. In contrast, in the A2780 CP70 line (p53 mutant) no induction of MDM2, p53, and p21 was observed. In contrast to the growth inhibition data, these results clearly demonstrate a p53-dependent cellular response to the pyrroles.

Compounds with *N*-phenylpyrrole or alkylidene barbituric acid groups have been identified as ‘frequent-hitters’ in HTS campaigns.^38^ With this in mind, it is likely that, despite the ability of these compounds to activate p53 dependent cellular processes, the modest growth inhibition seen for this series is the result of additional off-target activity.

## Conclusions

To date, the majority of potent MDM2–p53 inhibitors are highly selective for MDM2 over MDMX, and there are very few published inhibitors of MDM2 that retain significant potency for MDMX. The imidazole series, *e.g.* **1a**, is reported to have weak MDMX activity. The hydantoin derivative RO-5963 is reported to be a potent dual MDM2 and MDMX inhibitor, but with an unusual binding mode and cellular mechanism of action. We have identified a series of 1,2,5-triarylpyrroles that display MDM2–p53 and MDMX–p53 inhibitory activity in an cell-free ELISA MDM2–p53 binding assay, and are able to activate p53-dependent gene transcription in whole cells. Modeling studies suggest a binding mode which is consistent with other reported MDM2 inhibitors, and that offers insight into the structural requirements for the design of compounds able to inhibit both MDM2–p53 and MDMX–p53. However, the lack of p53-dependent growth inhibitory activity and the poor physicochemical properties for this series presents a substantial challenge for their further development as drugs; however, they represent interesting dual MDM2/MDMX ligands for both structural and mechanistic studies.

## Abbreviations

DCM: Dichloromethane
DIBAL-H: Di-isopropylaluminium hydride
DIPEA: Diisopropylethyl amine
DMF: *N*,*N*-Dimethylformamide
DMSO: Dimethyl sulfoxide
ELISA: Enzyme-linked immunosorbent assay
MDM: Murine double minute
NMO: *N*-Methylmorpholine-*N*-oxide
PCC: Pyridinium chlorochromate
PTSA: *p*-Toluenesulfonic acid
SAR: Structure–activity relationship
SRB: Sulforhodamine B
TFA: Trifluoroacetic acid
TFE: 2,2,2-Trifluoroethanol
THF: Tetrahydrofuran
TPAP: Tetrapropylammonium perruthenate
wt: Wild-type.
