# Supplementary material for: Diaryl- and triaryl-pyrrole derivatives: inhibitors of the MDM2–p53 and MDMX–p53 protein–protein interactions
Source: Medchemcomm. 2013 Jul 29;4(9):1297–304. doi: 10.1039/c3md00161j (PMC3777193; doi:10.1039/c3md00161j)
Supplement: Supplementary file 1 [file MD-004-c3md00161j-s001.pdf]

Electronic Supplementary Material (ESI) for Medicinal Chemistry Communications
This journal is © The Royal Society of Chemistry 2013

Diaryl- and Triaryl-pyrrole derivatives: inhibitors of the MDM2-p53 and MDMX-p53 protein-protein interactions.

Tim J. Blackburn[1], Shafiq Ahmed[2], Christopher R. Coxon[1], Junfeng Liu[2], Xiaohong Lu[2], Bernard T. Golding[1], Roger J. Griffin[1], Claire Hutton[2], David R. Newell[2], Stephen Ojo[1], Anna F. Watson[1], Andrey Zaytzev[1], Yan Zhao[2], John Lunec[2*], Ian R. Hardcastle[1*]

[1] Newcastle Cancer Centre, Northern Institute for Cancer Research and School of Chemistry, Bedson Building, Newcastle University, Newcastle, NE1 7RU, UK

[2] Newcastle Cancer Centre, Northern Institute for Cancer Research, Paul O'Gorman Building, Medical School, Framlington Place, Newcastle University, Newcastle, NE2 4HH, UK

SUPPORTING INFORMATION

EXPERIMENTAL

Determination of inhibition of the MDM2-p53 and MDMX-p53 interactions using binding assays (ELISA). Assays for MDM2-p53 inhibition were carried out as described previously.[1] Assays for MDMX-p53 inhibition were conducted using an similar method. A pCMV-XL5-MDMX cDNA construct (OriGene Technologies) was used for the *in vitro* coupled T7 transcription and rabbit reticulocyte lysate translation of MDMX, and a rabbit anti-MDMX antigen affinity-purified polyclonal antibody (Bethyl Laboratories Inc, via UK supplier Cambridge Bioscience, UK, Cat No. A300-287 A) was used for the ELISA.

Western blot analysis for p53 activation in intact cells

Western blot analysis of p53, MDM2, $p21^{WAF1}$ and actin proteins in cells treated with the MDM2-p53 antagonists was carried out as described previously.[1] Detection of α-tubulin on western blots with the Clone DM1A monoclonal antibody (Sigma-Aldrich, Dorset, UK) at 1:2000 dilution was used as an additional protein loading control.

Molecular modelling

PDB coordinates and electron density maps for 3LBK (MDM2) and 3LBK (MDM2) were imported into COOT (v0.7).[2] The ligands, **1b** in 3LBK (MDM2) and **1a** in 3LBJ (MDMX), respectively, were converted using the CCP4 'cprodrg' function.[3] Experimental ligands **4c**, **11c** and **11d** were built in 2D using the ligand builder function. Ligand **4c** was superimposed onto both **4a** and **4b**, using a graph map function, followed by minimisation *in situ* using the 'Sphere Refine' tool. The original ligand was deleted from the model and new coordinates for the modeled complex generated. Coordinates for ligands **11c** and **11d** were generated by their superimposition onto the model of **4c** using the same method.

This journal is © The Royal Society of Chemistry 2013

General Synthetic Methods

Reagents were purchased from fine chemicals vendors, and used as received unless otherwise stated. Solvents were purified and stored according to standard procedures. Petrol refers to that fraction in the boiling range 40-60 °C. THF refers to anhydrous tetrahydrofuran, obtained either by distillaton from sodium benzophenone, or from commercial sources. Melting points were obtained on a Stuart Scientific SMP3 apparatus and are uncorrected. Thin layer chromatography was performed using silica gel plates (Kieselgel 60F254; 0.2 mm), and visualized with UV light or potassium permanganate. Chromatography was conducted under medium pressure in glass columns or using a Biotage SP4 instrument in prepacked columns (FLASH+ Silica columns (40-63 μm, 60 Å). Proton (1H) and carbon (13C) nuclear magnetic resonance (NMR) spectra were recorded on a Bruker Spectrospin AC 300E (1H at 300 MHz, 13C at 75 MHz), a Jeol JNM-LA500 spectrometer (1H at 500 MHz, 13C at 125 MHz), or a Bruker Avance II 500 (1H at 500 MHz, 13C at 125 MHz) employing the solvent as internal standard. IR spectra were recorded on a Bio-Rad FTS 3000MX diamond ATR. Liquid Chromatography-Mass Spectrometry (LCMS) was carried out on a Micromass Platform instrument operating in positive and negative ion electrospray mode, employing a 50 x 4.6 mm C18 column (Waters Symmetry or Waters Atlantis) with 5 or 12 min gradient elution with 0.05% formic acid in methanol (10-90%), final compounds were ≥ 95% purity. Elemental analyses were performed by The School of Pharmacy, Analytical Facility, University of London, WC1N 1AX. Accurate masses were measured using a Finnigan MAR 95 XP or a Finnigan MAR 900 XLT at the EPSRC National Mass Spectrometry Service Centre (Chemistry Department, University of Wales, Swansea, Wales, SA2 8PP).

Synthesis

General Procedure A

To a mixture of the appropriate aniline (1.1 eq unless otherwise stated) of 1,2-dibenzoylethane (1.0 eq) and 2,2,2,-trifluoroethanol in a sealed microwave vial of appropriate capacity, was added trifluoroacetic acid (2.0 eq), drop-wise, under N_2 and then heated by microwave for 20 min at 150 ^{o}C. The resulting suspension was basified by addition of aqueous sodium hydroxide (1M) with stirring, filtered, and the solid washed with ethanol. The crude product **5** was dried *in vacuo*.

General Procedure B

To an appropriate capacity microwave vial was added the appropriate pyrrole **5** (1.0 eq) and anhydrous DMF and the vial was sealed. The mixture was cooled to -5 ^{o}C in an ice-salt bath, phosphorus oxychloride (3.0 eq) was added dropwise, under N_2 and heated by microwave 10 min at 70 ^{o}C. The mixture was poured over ice, basified with sodium hydroxide (1M), and heated to reflux for 1 h, then cooled to 0 ^{o}C and ethyl acetate (50

mL) added. The organic layer was washed with HCl (10%, 2 x 50 mL), water (2 x 50 mL), dried (sodium sulfate), and evaporated. Crude **6** was washed with ethanol and dried *in vacuo*.

General Procedure C

A mixture of 1-(4-halophenyl)-2,5-diphenyl-1*H*-pyrrole-3-carbaldehyde **6** (1 mol. eq.) and the appropriate barbituric acid (1.1 mol. eq.) in anhydrous EtOH was stirred at room temperature for 16 h, then concentrated *in vacuo*. Chromatography gave **4** as an orange solid.

General Procedure D

A mixture of 1-(4-halophenyl)-2,5-diphenyl-1*H*-pyrrole-3-carbaldehyde **6** (1 mol. eq.), the appropriate barbituric acid (1.1 mol. eq.) and glacial acetic acid (11 mL/mmol) was heated to 120 ^{o}C for 2 h, then filtered hot. The solid was washed with acetic acid (2 x 5 mL) and water (2 x 10 mL), and dried *in vacuo*, to give **4** as an orange solid.

General Procedure E

A solution of 1-(4-chlorophenyl)-2,5-diphenyl-1H-pyrrole-3-carbaldehyde **6a** (1 eq), the appropriate dicarbonyl compound (1.25 eq), piperidine (3 µL) and acetic acid (7 µL) in toluene (1.24 mL) was heated to reflux for 5 h. The precipitate was filtered. Chromatography (silica; ethyl acetate,methanol) gave **7** or **10** as a yellow solid.

General Procedure F

To dry DCM (8 mL per mmol **15**) at room temperature under nitrogen was added diethylzinc (4.1 eq, 1 M in hexanes). Diiodomethane (4.1 eq) was added dropwise at 0 ^{o}C and stirring continued for 10 minutes and a white suspension formed. The appropriate β-ketoester **15** (1 eq) was added dropwise and stirring was continued 30 min, then the appropriate aldehyde (1.05 eq) was added and the resulting pale yellow solution was stirred for 1 h, and quenched with sat. aqueous ammonium chloride and the organic phase was separated. The aqueous phase was extracted with Et_2O (2 x 20 mL), and the combined organic phases were washed with brine and dried (Na_2SO_4), and concentrated *in vacuo.*

The crude product (1 eq.) was dissolved in DCM (1.4 mL per mmol **19**) and pyridinium chlorochromate (PCC, 2.1 eq) was added at rt and the mixture stirred 18 h. In some cases additional PCC (1.05 eq.) was added. The mixture was filtered through a short pad of silica gel and the filtrate was concentrated *in vacuo* to give **16**.

General Procedure G

A mixture of 1,4-dicarbonyl compound **12** (1 eq.), aniline (1.2 eq.), *p*-toluene sulfonic acid (PTSA, 0.05 eq.) and toluene (0.94 mL per mmol **12**) was heated to reflux for 18 h. The mixture was cooled to rt, then filtered and washed with toluene through Celite™. The filtrate was concentrated *in vacuo.* Chromatography (silica; EtOAc, hexane) gave **13**.

General Procedure H

To a solution of pyrrole ester **18** (1 eq.) in dry DCM (14 mL per mmol **18**) at -78°C under nitrogen was added di-isopropylaluminium hydride (DIBAL-H, 3 eq., 1 M solution in hexane) and the resulting solution allowed to stir for 2 hours. The mixture was quenched by the dropwise addition of methanol and allowed to warm to room temperature, diluted with aqueous Rochelle's salt and stirred vigorously for 30 min, then was extracted with EtOAc (x 4). The combined organic phases were washed with brine, dried (Na_2SO_4), and concentrated *in vacuo*. Chromatography (silica; EtOAc, hexane) gave **19**.

General Procedure I

To a suspension of pyrrole alcohol **19** (1 eq.) and 4 Å molecular sieves (500 mg per mmol **19**) in dry DCM (2 mL per mmol **19**) was added *N*-methylmorpholine-*N*-oxide (NMO, 2 eq.) and the mixture stirred for 10 min. Tetrapropylammonium perruthenate (TPAP, 0.1 eq.) was added and the mixture stirred until TLC indicated the complete consumption of the starting material (ca. 30 min). The mixture was diluted with DCM and filtered and washed with ether thorough Celite™. The organic phase was washed with aq. sodium metabisulfite and brine. The aqueous layer was extracted with Et_2O (x 3). The combined organic extracts were dried ($MgSO_4$) and concentrated *in vacuo*. Chromatography (silica; EtOAc, hexane) gave **17**.

General Procedure J

A mixture of 1-(4-halophenyl)-2,5-disubstituted-1*H*-pyrrole-3-carbaldehyde **17** (1 mol. eq.), the appropriate barbituric acid (1.1 mol. eq.) and glacial acetic acid (11 mL/mmol) was heated to 120 °C for 2 h, then filtered hot. The solid was washed with acetic acid (2 x 5 mL) and water (2 x 10 mL), and dried *in vacuo*, to give **11** as an orange solid.

1-(4-Chlorophenyl)-2,5-diphenyl-1*H*-pyrrole (5a)

General Procedure A: 4-chloroaniline (0.24 g, 1.90 mmol), 1,2-dibenzoylethane (0.41 g, 1.72 mmol) and TFA (0.26 ml, 3.44 mmol) in TFE (20 mL) gave **5a** as a white solid (0.45 g, 79%). mp 224-226 °C; IR (cm^{-1}) 3026, 3031, 1676, 1593, 1487; ^1H NMR (300 MHz, $CDCl_3$) δ 6.49 (s, 2H, pyrrole-H), 6.96 (d, *J* = 8.7 Hz, 2H, -$C_2H_2C_2H_2C(Cl)$), 7.04-7.12 (m, 4H, Ar-H), 7.18-7.22 (m, 6H, ArH), 7.23 (m, 2H, ArH); ^{13}C NMR (75 MHz, $CDCl_3$) δ 110.6, 126.8, 128.3, 129.2, 130.5, 133.6, 136.4. LCMS (ES^+) m/z = 330 $[M+H]^+$.

This journal is © The Royal Society of Chemistry 2013

1-(4-Bromophenyl)-2,5-diphenyl-1*H*-pyrrole (5b)

General Procedure A: 4-bromoaniline (0.33 g, 1.90 mmol), 1,2-dibenzoylethane (0.41 g, 1.72 mmol) and TFA (0.26 ml, 3.44 mmol) in trifluoroethanol (20 mL) gave **5b** as a white solid (0.53 g, 83%). mp: 218-219 °C; IR (cm^{-1}): 3055, 1595, 1484, 1396; ^{1}H NMR (300 MHz, $CDCl_3$) δ 6.49 (s, 2H, pyrrole-H), 6.91 (d AB, *J* = 8.7 Hz, 2H, -$C_2H_2C_2H_2C$(Br)), 7.05-7.10 (m, 4H, ArH), 7.18-7.23 (m, 6H, ArH), 7.37 (d, *J* = 8.7 Hz, 2H, -$C_2H_2C_2H_2C$(Br)). ^{13}C NMR (75 MHz, $CDCl_3$) δ 99.9, 110.5, 126.7, 128.2, 129.1, 130.7, 132.1, 133.4, 136.2. LCMS (ES^+) m/z = 374, 376 $[M+H]^+$.

4-(2,5-Diphenyl-1*H*-pyrrol-1-yl)benzonitrile (5c)

General Procedure A: 4-aminobenzonitrile (0.054 g, 0.46 mmol), 1,2-dibenzoylethane (0.1 g, 0.42 mmol,) and TFA (0.96 g, 0.84 mmol) in TFE gave **5c** as a white solid. (0.049 g, 37%). mp 247-249 °C; ^{1}H NMR (300 MHz, d_6-DMSO) δ 7.81 (d, *J* = 8.5 Hz, 2 H, Ar-H), 7.03 (d, *J* = 8.10 Hz, 2 H, Ar-H), 7.22 (s, 10 H, Ar-H), 6.51 (s, 2 H, pyrrole).

1-(4-(*t*-Butyl)phenyl)-2,5-diphenyl-1*H*-pyrrole (**5d**)

General Procedure A: 4-*t*-butylaniline (0.051 g, 0.46 mmol), 1,2-dibenzoylethane (0.1 g, 0.42 mmol), TFA (0.96 g, 0.84 mmol) gave **5d** as a white solid. (124 mg ; 85%): mp 246-248 °C; ^{1}H NMR (300 MHz, d_6-DMSO) δ 7.33 (d, *J* = 8.5 Hz, 2 H, Ar-H), 7.01 (d, *J* = 8.5 Hz, 2 H, Ar-H), 7.13 (m, 10 H, Ar-H), 6.46 (s, 2 H, pyrrole), 3.33 (d, *J* = 7.1 Hz, 9 H, *t*-Butyl); LCMS (ES^+) *m/z* = 368.3 $[M+H]^+$.

1-(4-Nitrophenyl)-2,5-diphenyl-1*H*-pyrrole (5e)

General Procedure A: 4-nitroaniline (0.064 g, 0.46 mmol), 1,2-dibenzoylethane (0.1 g, 0.42 mmol), TFA (0.96 g, 0.84 mmol) in TFE gave **5e** as a white solid (39 mg, 27%): mp 244-246 °C; ^{1}H NMR (300 MHz, d_6-DMSO) δ 8.2 (d, *J* = 8.9, 2 H, Ar-H), 7.3 (d, *J* = 8.9, 2 H, Ar-H), 7.5 (m, 10 H, Ar-H), 6.54 (s, 2 H, pyrrole).

1-(4-Methoxyphenyl)-2,5-diphenyl-1*H*-pyrrole (5f)

General Procedure A: 4-methoxyaniline (0.057 g, 0.46 mmol), 1,2-dibenzoylethane (0.1 g, 0.42 mmol), TFA (0.96 g, 0.84 mmol) in TFE gave **5f** as a white solid (117 mg, 86%). mp 227-229 °C.

Methyl 1-(4-bromophenyl)-5-(*tert*-butyl)-2-phenyl-1*H*-pyrrole-3-carboxylate (19b)

General Procedure A: **16c** (0.20 g, 0.72 mmol), 4-bromoaniline (0.622 g, 3.6 mmol), and TFA (0.1 ml, 1.44mmol) gave **19b** as white flaky solid (889 mg; 60%). IR (cm^{-1}) 2967, 2943, 1715, 1483; ^{1}H NMR (300 MHz) δ 1.12 (s, 9H, *t*-Bu), 3.58 (s, 3H, CH_3), 6.51 (s, 1H, 4-H), 6.95-7.05 (m, 4H, Ar-H), 7.08-7.12 (m, 3H, Ar-H), 7.24-

7.31 (m, 2H, Ar-H); ^{13}C NMR (100 MHz) δ 31.5, 33.2, 50.9, 107.7, 113.0, 122.8, 127.6, 128.0, 131.5, 131.6, 132.3, 132.7, 139.3, 141.1, 144.2, 165.5; LCMS (ES$^+$) m/z = 412.00 [M+H]$^+$.

Methyl 5-(tert-butyl)-1-(4-chlorophenyl)-2-phenyl-1H-pyrrole-3-carboxylate (19c)

General Procedure A: **16c** (0.150 g, 0.54 mmol), 4-chloroaniline (0.138g, 1.08mmol), and TFA (0.08 ml, 1.08mmol) gave **19c** as a white flaky solid (0.105 g, 52%): IR (cm^{-1}) 2965, 1709, 1487, 1229; ^{1}H NMR (300 MHz) δ 1.19 (s, 9H, *t*-Bu), 3.68 (s, 3H, CH_3), 6.61 (s, 1H, 4-H), 7.08-7.24 (m, 9H, Ar-H); ^{13}C (100 MHz) δ 31.5, 33.2, 127.6, 127.9, 128.6, 131.5, 132.4, 144.2; LCMS (ES$^+$) m/z = 367 [M+H]$^+$;

1-(4-Chlorophenyl)-2,5-diphenyl-1*H*-pyrrole-3-carbaldehyde (6a)

General Procedure B: **5a** (0.20 g, 0.6 mmol), phosphorous oxychloride (0.16 mL, 1.8 mmol) in DMF (8 mL) gave **6a** as a white solid (0.18 g, 84%). mp 222-223 ^{o}C; IR (cm^{-1}) 3099, 3073, 3049, 2833, 2749, 1649, 1601. ^{1}H NMR (300 MHz, $CDCl_3$) δ 6.91 (d, *J* = 8.7 Hz, 2H, Ar-H), 6.99 (s, 1H, pyrrole-H), 7.06-7.14 (m, 2H, Ar-H), 7.17-7.23 (m, 4H, Ar-H), 7.26-7.29 (m, 4H, Ar-H), 7.32-7.36 (m, 2H, Ar-H), 9.72 (s, 1H, CHO); ^{13}C NMR (75 MHz, $CDCl_3$) δ 98.1, 105.3, 112.9, 116.4, 119.6, 122.5, 123.8, 125.4, 126.0, 127.1, 128.3, 128.6, 129.3, 130.3, 131.6, 136.4, 194.8. LCMS (ES$^+$) m/z = 358 [M+H]$^+$.

1-(4-Bromophenyl)-2,5-diphenyl-1*H*-pyrrole-3-carbaldehyde (6b)

General Procedure B: 1-(4-bromophenyl)-2,5-diphenyl-1*H*-pyrrole **5b** (0.225 g, 0.6 mmol), phosphorous oxychloride (0.16 mL, 1.8 mmol) in DMF (8 mL) gave **6b** as a cream solid (0.16 g, 67%). mp 193-194 ^{o}C; IR (cm^{-1}) 3066, 3037, 2826, 2743, 1739, 1651; ^{1}H NMR (300 MHz, $CDCl_3$) δ 6.84 (d, *J* = 8.5 Hz, 2H, ArH), 6.98 (s, 1H, pyrrole-H), 7.09-7.14 (m, 2H, Ar-H), 7.18-7.27 (m, 4H, Ar-H), 7.30-7.38 (m, 6H, Ar-H), 9.72 (s, 1H, CHO); ^{13}C NMR (75 MHz, $CDCl_3$) δ 100.0, 118.9, 119.3, 119.6, 128.5, 128.6, 129.1, 129.3, 130.5, 131.5, 132.4, 134.6, 135.4, 140.9, 145.1, 148.5, 191.9. LCMS (ES$^+$) m/z = 402, 404 [M+H]$^+$.

4-(3-Formyl-2,5-diphenyl-1*H*-pyrrol-1-yl)benzonitrile (6c)

General Procedure B: **5c** (0.096 g, 0.3 mmol), phosphorus oxychloride (0.08 mL, 0.9 mmol) in DMF (4 mL) gave **6c** as a cream solid (0.084 g, 80%) mp 259-261 ^{o}C; ^{1}H NMR (300 MHz, d_6-DMSO) δ 9.57 (s, 1H, CHO), 6.90 (s, 1H, pyrrole), 7.80 (d, *J* = 8.6, 2H, Ar-H), 7.34 (d, *J* = 8.5, 2H, Ar-H), 7.24 (m, 10H, Ar-H).

1-(4-(*t*-Butyl)phenyl)-2,5-diphenyl-1*H*-pyrrole-3-carbaldehyde (6d)

General Procedure B: **5d** (0.11 g, 0.3 mmol,) and phosphorus oxychloride (0.08 mL, 0.9 mmol) in DMF (4 mL) gave **6d** as a cream solid. (0.051 mg, 48%). mp 174-176 ^{o}C; ^{1}H NMR (300 MHz, d_6-DMSO) δ 9.53 (s, 1H, CHO), 6.84 (s, 1H, Pyrrole-H), 7.08 (d, *J* = 8.8 Hz, 2H, Ar-H), 6.81 (d, *J* = 8.9 Hz, 2H, Ar-H), 7.24 (m, 10H, Ar-H), 3.34 (s, 9H, *t*-Butyl).

1-(4-Nitrophenyl)-2,5-diphenyl-1*H*-pyrrole-3-carbaldehyde (6e)

General Procedure B: **5e** (0.1 g, 0.3 mmol), phosphorus oxychloride (0.08 mL, 0.9 mmol,) in DMF (4 mL) gave **6e** as a cream solid. (24.3 mg, 22%). mp 208-209 °C; ^{1}H NMR (300 MHz, d_6-DMSO) δ 6.92 (s, 1H, pyrrole-H), 7.31 (m, 10H, Ar-H), 7.41 (d, *J* = 8.9 Hz, 2H, Ar-H), 8.13 (d, *J* = 8.9 Hz, 2H, Ar-H), 9.58 (s, 1H, CHO).

1-(4-Methoxyphenyl)-2,5-diphenyl-1*H*-pyrrole-3-carbaldehyde (6f)

General Procedure B: **5f** (0.098 g, 0.3 mmol), phosphorus oxychloride (0.08 mL, 0.9 mmol,) in DMF (4 mL) gave **6f** as a white solid. (90 mg, 79%). mp 180-181 °C; ^{1}H NMR (300 MHz, d_6-DMSO) δ 3.68 (s, 3H, OCH_3), 6.81 (d, *J* = 8.9 Hz, 2H, N-Ar), 7.08 (d, *J* = 8.9 Hz, 2H, N-Ar), 7.25 (m, 10H, Ph), 9.53 (s, 1H, CHO). LCMS (ES^+) m/z = 353.2 $[M+H]^+$.

5-((1-(4-Chlorophenyl)-2,5-diphenyl-1*H*-pyrrol-3-yl)methylene)-2-thioxodihydropyrimidine-4,6(1*H*,5*H*)-dione (4c)

General Procedure C: **6a** (100 mg, 0.28 mmol), thiobarbituric acid (44.7 mg, 0.31 mmol), EtOH (3 mL) gave **4c** (127 mg, 95%). mp 250-251 °C; ^{1}H NMR (300 MHz, d_6-DMSO) 12.2 (s, 1 H, NH), 12.1 (s, 1 H, NH) 8.01 (s, 1 H, vinyl), 7.32 (d, *J* = 8.70 Hz, 2 H, Ar-H), 7.18 (d, *J* = 8.7 Hz, 2 H, Ar-H), 7.27 (m, 11 H, Ar-H).

5-((1-(4-Chlorophenyl)-2,5-diphenyl-1*H*-pyrrol-3-yl)methylene)pyrimidine-2,4,6(1*H*,3*H*,5*H*)-trione (4m)

General Procedure D: **6a** (100 mg, 0.28 mmol), barbituric acid (42 mg, 0.26 mmol), glacial acetic acid (3 mL) gave **4m** (98.7 mg, 75%). mp 364-366 °C; 1H NMR (300 MHz, d_6-DMSO) δ 11.1 (s, 1 H, NH), 11.0 (s, 1 H, NH), 8.01 (s, 1 H, vinyl), 7.29 (m, 11 H, Ar-H), 7.19 (d, *J* = 8.7 Hz, 2 H, Ar-H), 7.34 (d, *J* = 8.7 Hz, 2 H, Ar-H); LCMS (ES^+) m/z = 468.20 $[M+H]^+$.

5-((1-(4-Bromophenyl)-2,5-diphenyl-1*H*-pyrrol-3-yl)methylene)-2-pyrimidine-2,4,6(1*H*,3*H*,5*H*)-trione (4n)

General Procedure D: **6b** (100 mg, 0.24 mmol), barbituric acid (36 mg, 0.26 mmol), glacial acetic acid (3 mL) gave **4n** (102 mg, 87%). mp 366-368 °C; ^{1}H NMR (300 MHz, d_6-DMSO) δ 7.12 (d, *J* = 8.7, 2H, Ar-H) , 7.47 (d, *J* = 8.7, 2H, Ar-H), 7.27 (s, 1H, pyrrole) , 7.31 (m, 10H, Ar-H), 8.01 (s, 1H, vinyl), 11.01 (s, 1H), 11.06 (s, 1H, NH); LCMS (ES^+) m/z = 512 [M+ H].

5-((1-(4-Methoxyphenyl)-2,5-diphenyl-1*H*-pyrrol-3-yl)methylene)pyrimidine-2,4,6(1*H*,3*H*,5*H*)-trione (4o)

General Procedure D: 1-(4-methoxyphenyl)-2,5-diphenyl-1H-pyrrole-3-carbaldehyde **6f** (100 mg, 0.26 mmol), barbituric acid (41.5 mg, 0.29 mmol, 1.1 eq), glacial acetic acid (3 mL) gave **4o** as a bright yellow solid (74 mg, 74%). mp 347-349 °C; ^{1}H NMR (300 MHz, d_6-DMSO) δ 1.18 (s, 3 H, OMe), 7.19 (m, 10 H, phenyl), 7.06 (d, *J* =

7.63, 2 H, N-Ar), 7.29 (s, 1 H, pyrrole), 7.36 (d, *J* = 7.63, 2 H, N-Ar), 8.01 (s, 1 H, vinyl), 11.03 (s, 1 H, NH), 11.08 (s, 1 H, NH);

5-((1-(4-(*t*-Butyl)phenyl)-2,5-diphenyl-1*H*-pyrrol-3-yl)methylene)pyrimidine-2,4,6(1*H*,3*H*,5*H*)-trione (4p)

General Procedure D: **6d** (100 mg, 0.26 mmol), barbituric acid (42 mg, 0.31 mmol), glacial acetic acid (3 mL) gave **4p** as bright yellow solid (16.2 mg, 13%). mp >320°C; ^{1}H NMR (300 MHz, d_6-DMSO) δ 3.34 (s, 9 H, t-butyl), 7.25 (m, 11 H, Ar-H & pyrrole), 7.06 (d, *J* = 8.54, 2 H, Ar-H), 7.36 (d, *J* = 7.71, 2 H, Ar-H), 8.01 (s, 1 H, vinyl), 11.03 (s, 1 H), 11.08 (s, 1 H, NH); LCMS (ES$^+$) m/z = 490 [M+H]$^+$; CHN C, 76.05; H, 5.56; N, 8.58; found C 75.53, H 5.52, N 8.84;

4-(2,5-Diphenyl-3-((2,4,6-trioxotetrahydropyrimidin-5(2*H*)-ylidene)methyl)-1*H*-pyrrol-1-yl)benzonitrile (4q)

General Procedure D: **6c** (100 mg, 0.29 mmol), barbituric acid (42 mg, 0.31 mmol), glacial acetic acid (3 mL) gave **4q** as bright yellow solid (94.0 mg, 70%). mp 368-370 °C; ^{1}H NMR (300 MHz, *d*$_6$-DMSO) δ 7.23 (m, 11 H, Ar-H, pyrrole), 7.39 (d, *J* = 8.96, 2 H, Ar-H), 7.79 (d, *J* = 8.48, 2 H, Ar-H) , 8.01 (s, 1 H, vinyl), 11.08 (s, 1 H, NH), 11.12 (s, 1 H, NH); LCMS (ES$^-$) m/z = 457 [M-H]$^-$.

4-(2,5-Diphenyl-3-((2-thioxodihydropyrimidine-4,6(1*H*,5*H*)-dione)methyl)-1*H*-pyrrol-1-yl)benzonitrile (4r)

General Procedure D: **6c** (100 mg, 0.29 mmol), thiobarbituric acid (46 mg, 0.32 mmol), glacial acetic acid (3 mL) gave **4r** as bright yellow solid (119 mg, 86%). mp 334-336 °C (dec); ^{1}H NMR (300 MHz, *d*$_6$-DMSO) δ 7.14 (dd, *J* = 6.51, 3.00 Hz, 3H), 7.28 (ddd, *J* = 9.33, 6.48, 1.62 Hz, 5H) , 7.42 (dd, *J* = 9.32, 3.79 Hz, 3H), 7.39 (d, *J* = 8.46 Hz, 2H, Ar-N), 7.80 (d, *J* = 8.56 Hz, 2H, Ar-CN), 8.04 (s, 1H), 8.06 (s, 1H, vinyl), 12.22 (s, 1H, NH ex), 12.24 (s, 1H, NH ex) ; LCMS (ES$^+$) m/z = 475.4 [M+H]$^+$.

5-((1-(4-Nitrophenyl)-2,5-diphenyl-1*H*-pyrrol-3-yl)methylene)pyrimidine-2,4,6(1*H*,3*H*,5*H*)-trione (4s)

General Procedure D: **6e** (100 mg, 0.29 mmol), barbituric acid (42 mg, 0.31 mmol), glacial acetic acid (3 mL) gave **4s** as bright yellow solid (94 mg, 70%). mp 368-370 °C; ^{1}H NMR (300 MHz, *d*$_6$-DMSO) δ 7.23 (m, 11 H, Ar-H, pyrrole), 7.39 (d, *J* = 8.96, 2 H, Ar-H), 7.79 (d, *J* = 8.48, 2 H, Ar-H) , 8.01 (s, 1 H, vinyl), 11.08 (s, 1 H, NH), 11.12 (s, 1 H, NH); LCMS (ES$^-$) m/z = 457 [M-H]$^-$.

5-((1-(4-Nitrophenyl)-2,5-diphenyl-1*H*-pyrrol-3-yl)methylene)-2-thioxodihydropyrimidine-4,6(1*H*,5*H*)-dione (4t)

General Procedure D: **6e** (100 mg, 0.29 mmol), thiobarbituric acid (43 mg, 0.30 mmol), glacial acetic acid (3 mL) gave **4t** as bright yellow solid (107 mg, 79%). mp 333-334 °C; ^{1}H NMR (300 MHz, *d*$_6$-DMSO) δ 7.16 (dd, *J* = 6.53, 2.94 Hz, 3H, phenyl), 7.29 (m, 5H, phenyl, Ar-N), 7.44 (dd, *J* = 13.77, 8.10 Hz, 5H, phenyl),8.06 (d, *J* =

Electronic Supplementary Material (ESI) for Medicinal Chemistry Communications
This journal is © The Royal Society of Chemistry 2013

11.70 Hz, 2H, Ar-NO_2), 8.13 (s, 1H), 8.16 (s, 1H, vinyl), 12.24 (s, 1H, NH ex), 12.25 (s, 1H, NH ex); LCMS (ES^+) m/z = 495.3 $[M+H]^+$. Anal. Calcd. for $C_{27}H_{18}N_4O_4S$: req.uires C, 65.58; H, 3.67; N, 11.32%; found: C, 65.56; H, 3.60; N, 11.41%

5-((1-(4-Chlorophenyl)-2,5-diphenyl-1*H*-pyrrol-3-yl)methylene)-1,3-diethyl-2-thioxodihydropyrimidine-4,6(1*H*,5*H*)-dione (4u)

General Procedure C: **6a** (0.195 g, 0.54 mmol), 1,3-diethyl-2-thiobarbituric acid (0.12 g, 0.6 mmol), EtOH (6 mL). Chromatography (silica; DCM) gave **4u** as an orange solid (0.26 g, 89%). mp 272-273 ^oC. IR (cm^{-1}) 3171, 3082, 2979, 2927, 2162, 2029, 1694, 1661; ^1H NMR (300 MHz, $CDCl_3$) δ 1.32 (t, *J* = 7.0 Hz, 6H, CH_3), 4.58 (q, *J* = 7.0 Hz, 4H, 2 x CH_2), 6.89 (d, *J* = 8.7 Hz, 2H, -$C_2H_2C_2H_2C(Cl)$), 7.13-7.22 (m, 6H, Ar-H), 7.25-7.27 (d, *J* = 8.7 Hz, 2H, -$C_2H_2C_2H_2C(Cl)$), 7.34-7.41 (m, 4H , Ar-H), 8.09 (s, 1H, pyrrole), 8.40 (s, 1H, -C=CH-). ^{13}C NMR (75 MHz, $CDCl_3$) δ 12.7, 12.9, 43.6, 44.1, 112.3, 114.4, 121.2, 128.1, 128.6, 128.7, 129.5, 129.6, 129.7, 129.9, 131.7, 132.0, 134.6, 136.6, 137.89, 149.9, 152.9, 159.7, 162.2, 179.6; HRMS (EI): $C_{31}H_{26}ClN_3O_2S$ calcd. m/z 540.1507 $[M]^+$; found m/z 540.1507 $[M]^+$. .

5-((1-(4-Bromophenyl)-2,5-diphenyl-1*H*-pyrrol-3-yl)methylene)-1,3-diethylpyrimidine-2,4,6(1*H*,3*H*,5*H*)-trione (4v)

General Procedure C: **6b** (0.07 g, 0.174 mmol) and *N*,*N*-diethylbarbituric acid (0.035 g, 0.191 mmol) in anhydrous EtOH (2 mL). Chromatography (silica; 75-100% DCM, petrol) gave **4v** as a yellow solid (0.051 g, 52%); mp 285-286 ^oC. λ_{max} (CH_3OH)/nm = 406. IR: 3167, 3083, 2924, 2851, 1722, 1657, 1542 cm^{-1}; ^1H NMR: (300 MHz, $CDCl_3$) δ 1.25 (t, *J* = 7.1 Hz, 6H, CH_3), 4.04 (q, *J* = 7.1 Hz, 4H, CH_2), 6.83 (d AB, *J* = 8.7 Hz, 2H, -$C_2H_2C_2H_2C(Br)$), 7.13-7.16 (m, 4H, ArH), 7.24-7.27 (m, 4H, ArH), 7.32-7.40 (m, 4H, ArH), 8.07 (s, 1H, pyrrole H), 8.39 (s, 1H, C=CH-). ^{13}C NMR: (75 MHz, $CDCl_3$) δ 16.94, 27.38, 103.76, 107.26, 113.94, 118.91, 123.17, 124.89, 128.19, 128.62, 129.62, 131.95, 134.81, 136.21, 150.21, 152.11, 158.75, 165.38. HRMS (EI): $C_{31}H_{26}BrN_3O_3$ Calcd.m/z = 567.1158 $[M]^+$, obsd m/z = 567.1178 $[M]^+$. Anal. Calcd. for $C_{31}H_{26}BrN_3O_3$: C, 65.49; H, 4.57; N, 7.39%. Found: C, 65.87; H, 4.39; N, 7.35%.

5-((1-(4-Bromophenyl)-2,5-diphenyl-1*H*-pyrrol-3-yl)methylene)-1,3-diethyl-2-thioxodihydropyrimidine-4,6(1*H*,5*H*)-dione (4w)

General Procedure C: **6b** (0.20 g, 0.497 mmol), 1,3-diethyl-2-thiobarbituric acid (0.11 g, 0.547 mmol), EtOH (6 mL). Chromatography (silica; DCM) gave **4w** as an orange solid (0.238 g, 82%). mp 271-272 ^oC. IR (cm^{-1}) 3171, 3081, 2978, 2928, 2160, 1693, 1660, 1533; ^1H NMR (300 MHz, $CDCl_3$) δ 1.32 (t, *J* = 6.9 Hz, 6H, CH_3), 4.58 (q, *J* = 6.9 Hz, 4H, 2 x CH_2), 6.85 (d, *J* = 8.7 Hz, 2H, -$C_2H_2C_2H_2C(Br)$), 7.13-7.17 (m, 4H, Ar-H), 7.25-7.27 (m, 2H, Ar-H), 7.34-7.41 (m, 6H, Ar-H), 8.09 (s, 1H, pyrrole proton), 8.40 (s, 1H, =C-CH-); ^{13}C NMR (75 MHz, $CDCl_3$) δ 12.7, 12.9, 43.6, 44.1, 112.3, 114.5, 121.2, 122.6, 128.1, 128.6, 128.7, 129.6, 129.7, 130.2, 131.7, 132.0,

Electronic Supplementary Material (ESI) for Medicinal Chemistry Communications
This journal is © The Royal Society of Chemistry 2013

132.5, 137.1, 137.9, 149.8, 152.9, 159.7, 162.1, 179.7. HRMS (EI): $C_{31}H_{26}{}^{79}BrN_3O_2S$ calcd. m/z 584.1002 $[M]^+$; found: m/z 584.0998 $[M]^+$. CHN

5-((1-(4-Chlorophenyl)-2,5-diphenyl-1H-pyrrol-3-yl)methylene)-3-methyl-2-thioxodihydropyrimidine-4,6(1*H*,5*H*)-dione (4x)

General Procedure D: **6a** (0.086 g, 0.24 mmol), 4,6-dihydroxy-1-methylpyrimidine-2(1*H*)-thione (0.042 g, 0.26 mmol), glacial acetic acid (3 mL). Chromatography (silica; DCM) gave a mixture of regioisomers **4x** as an orange solid (0.065 g, 53%). mp: 316-317 °C; IR (cm^{-1}) 3141, 3078, 2920, 2850, 2031, 2011, 1977, 1693, 1649. ^{1}H NMR (300 MHz, d_6-DMSO) δ 3.50 & 3.59 (2s, 2x3H, CH_3), 7.16-7.20 (d, J = 8.7, 2H, -$C_2H_2C_2H_2C(Cl)$), 7.22-7.34 (m, 6H, Ar-H), 7.35-7.39 (m, 3H, Ar-H), 7.40-7.45 (m, 3H, Ar-H), 8.04-8.07 (2 x s, 1H, C=CH-), 8.08 (s, 1H, pyrrole H), 12.38 (s, 1H, N*H*). ^{13}C NMR (75 MHz, d_6-DMSO) δ 35.1, 99.5, 111.8, 115.1, 121.7, 123.5, 124.7, 127.9, 128.5, 128.7, 129.1, 130.8, 132.0, 132.9, 138.3, 149.5, 156.5, 157.3, 185.8; LCMS m/z = 498 (ES^+). Anal. Calcd. for $C_{28}H_{20}ClN_3O_2S$ + 0.2 H_2O: C, 67.05; H, 4.10; N, 8.38%. Found: C, 67.45; H, 4.35; N, 7.99%.

5-((1-(4-Bromophenyl)-2,5-diphenyl-1H-pyrrol-3-yl)methylene)-1-methylpyrimidine-2,4,6(1*H*,3*H*,5*H*)-trione (4y)

General Procedure C: **6b** (0.07 g, 0.174 mmol), *N*-methylbarbituric acid (0.025 g, 0.191 mmol), EtOH (2 mL). Chromatography (silica; DCM - 1% MeOH/DCM) gave a mixture of regioisomers **4y** as a yellow solid (0.06 g, 60%). R_f = 0.34 and 0.41 (5:95 MeOH:DCM); IR (cm^{-1}) 695, 755, 790, 839, 961, 1028, 1068, 1138, 1189, 1306, 1371, 1443, 1483, 1538, 1654, 1684, 2850, 2920, 3065, 3167; ^{1}H NMR (300 MHz, $CDCl_3$) δ 3.34 and 3.40 (s, 3H, CH_3), 6.90 (d, J = 8.5, 2H, -$C_2H_2C_2H_2C(Br)$), 7.12-7.16 (m, 4H, Ar-H), 7.25-7.27 (m, 2H, ArH), 7.33-7.41 (m, 6H, Ar-H), 7.85 (s, 1H, NH), 8.09 (s, 1H, pyrrole H), 8.36 and 8.41 (s, 1H, C=CH). ^{13}C NMR (75 MHz, $CDCl_3$) δ 28.3, 100.4, 111.1, 114.5, 121.1, 128.1, 128.6, 128.7, 129.5, 129.6, 129.7, 130.5, 130.5, 130.9, 131.1, 130.2, 131.6, 131.6, 132.0, 132.5, 137.8, 141.8, 149.3, 149.7, 150.6, 159.4; LCMS (ES^+)m/z = 526, 528. HRMS (EI): m/z Calcd. for ion: 525.0688 $[M]^+$. Found: 525.0685 $[M]^+$. CHN.

5-((1-(4-Bromophenyl)-2,5-diphenyl-1H-pyrrol-3-yl)methylene)-3-methyl-2-thioxodihydropyrimidine-4,6(1H,5H)-dione (4z)

General Procedure D: **6b** (0.10 g, 0.25 mmol) and 4,6-dihydroxy-1-methylpyrimidine-2(1*H*)-thione (0.043 g, 0.27 mmol), glacial acetic acid (3 mL) gave **4z** as an orange solid (0.108 g, 81%). IR (cm^{-1}) 3138, 3076, 2928, 2164, 2069, 1976, 1695, 1649; ^{1}H NMR (300 MHz, DMSO) δ 3.50 and 3.59 (2 x s, 3H, CH_3), 7.18 (d, J = 8.6, 2H, $C_2H_2C_2H_2C(Br)$), 7.30-7.31 (m, 6H, Ar-H), 7.42-7.43 (m, 3H, Ar-H), 7.49-7.52 (m, 3H, Ar-H), 8.04 (1H, C=CH-), 8.08 (s, 1H, pyrrole H), 12.37 (br s, 1H, NH); ^{13}C NMR (75 MHz, DMSO) δ 32.3, 99.4, 110.5, 118.7, 121.1, 123.7, 126.4, 127.8, 128.6, 128.6, 129.1, 131.1, 132.1, 132.3, 132.8, 143.2, 150.1, 155.2, 158.8, 181.3. LCMS (ES^+) m/z = 542, 544. CHN.

5-((1-(4-Bromophenyl)-2,5-diphenyl-1*H*-pyrrol-3-yl)methylene)-2,2-dimethyl-1,3-dioxane-4,6-dione. (7)

General Procedure E: **6b** (100 mg, 0.248mmol), Meldrum's acid **8** (44.7 mg, 0.31 mmol). The reaction mixture was diluted with EtOAc (20 mL) and washed with saturated $NaHCO_3$ (2 x 10 mL), dried (Na_2SO_4) and concentrated *in vacuo*. Chromatography (5-40% EtOAc, hexane) gave **7** as a yellow solid (93 mg, 49%). mp 220 ^{o}C, IR (cm^{-1}) 2958, 1712, 1608, 1545; ^{1}H NMR (500 MHz, $CDCl_3$) δ 1.69 (6H, s, $(CH_3)_2$), 6.70-6.74 (2H, m, Ar-H), 7.05-7.06 (4H, m, Ar-H), 7.16-7.17 (2H, m, Ar-H), 7.26-7.45 (5H, m, Ar-H), 7.77 (1H, s, 4-H), 8.18 (1H, s, 3-CH); ^{13}C NMR (125MHz, $CDCl_3$) δ 14.1, 21.1, 22.6, 27.4, 29.7, 31.6, 60.4, 103.6, 106.8, 113.0, 119.1, 122.2, 127.7, 128.2, 128.5, 128.7, 129.0, 129.3, 129.8, 131.0, 132.2, 136.4, 137.5, 131.4, 148.5, 150.9, 161.1, 164.4, 171.1. LCMS (ES^{+}) m/z = 472 [M+H]$^{+}$;

5-((1-(4-Bromophenyl)-2,5-diphenyl-1*H*-pyrrol-3-yl)methyl)-2,2-dimethyl-1,3-dioxane-4,6-dione. (9)

To a suspension of **7** (50 mg; 0.095 mmol) in MeOH (0.6 mL) at 0°C was added $NaBH_4$ (3.6 mg; 0.19mmol), and mixture was allowed to warm to rt with stirring for 30 minutes. HCl (1M; 0.5mL) was added and the precipitate filtered, and washed with water giving **9** as a white solid (30 mg; 60%); mp 159 ^{o}C; 2023, 1748, 1490; ^{1}H NMR (300 MHz, $CDCl_3$) δ 7.25-7.28 (6H, m, Ar-H), 7.11-7.21 (4H, m, Ar-H), 6.97 (2H, d, *J* = 7.9 Hz, Ar-H), 6.77 (2H, d, *J* = 8.6 Hz, Ar-H), 6.42 (1H, s, 4-H), 3.62 (1H, t, *J* = 5.1 Hz, CH), 3.32 (2H, d, *J* = 5.1 Hz, 3-CH_2), 1.61 (3H, s, CH_3), 1.60 (3H, s, CH_3): δ_C (75MHz, $CDCl_3$) 24.2, 27.4, 28.9, 29.9, 47.8, 105.1, 110.7, 118.9, 121.0, 126.8, 127.7, 128.3, 128.9, 130.6, 131.5, 131.9, 132.4, 133.2, 134.2, 134.9, 138.5, 165.6. LCMS (ES^{+}) m/z = 364.

Dimethyl 2-((1-(4-chlorophenyl)-2,5-diphenyl-1H-pyrrol-3-yl)methylene)malonate (10a)

General Procedure E: **6a** (50 mg, 0.140 mmol), dimethyl malonate (20 µl, 0.18 mmol). Chromatography (30% EtOAc, Petrol) gave **10a** as a white solid (47 mg, 71%); mp 228.6 ^{o}C; IR (cm^{-1}) 3060, 2950, 2361, 1731; ^{1}H NMR (500MHz, $CDCl_3$) δ 3.76 (s, 3H, O-CH_3), 3.93 (s, 3H, O-CH_3), 6.54 (s, 1H, Pyrrole), 6.83-6.80 (m, 2H, Ar-H), 7.09-7.02 (m, 4H, Phenyl), 7.14-7.19 (m, 2H, Ar-H), 7.23-7.20 (m, 3H, Ar-H), 7.32-7.27 (m, 3H, Ph), 7.60 (s, 1H, 3-CH); ^{13}C NMR (125MHz, d_8-THF) δ 52.12, 52.40, 109.17, 118.22, 121.98, 128.18, 129.12, 129.14, 129.26, 129.89, 131.31, 131.47, 132.42, 133.17, 134.39, 136.17, 137.74, 138.00, 141.90, 165.59, 168.23; LCMS (ES^{+}) m/z = 472.18 [M+H^{+}]; HRMS**:** $C_{28}H_{22}O_4NClNa$ [M+Na]$^{+}$ Calculated: 494.1130, Found**:** 494.1121

2-((1-(4-Chlorophenyl)-2,5-diphenyl-1H-pyrrol-3-yl)methylene)malonic acid (10b) and (E)-3-(1-(4-chlorophenyl)-2,5-diphenyl-1H-pyrrol-3-yl) acrylic acid (10f)

General Procedure E: **6a** (50 mg, 0.140 mmol), malonic acid (18 mg, 0.175 mmol). Chromatography (30% EtOAc, petrol then 1% MeOH, EtOAc) gave **10b** as a white solid (30 mg, 48%). mp 302 ^{o}C (dec); IR (cm^{-1})

3058, 2923, 2183, 1628, 1493; LCMS (ES$^+$) m/z = 444 $[M+H]^+$; HRMS: $C_{26}H_{17}NO_4ClNa$ $[M-H]^+$ Calculated: 442.0852, Found: 442.0857.

and **10f** (17 mg, 30%) mp 302 ^oC (dec) IR (cm^{-1}) 3054, 2361, 1676, 1594, 1496; ^{1}H NMR (500MHz, DMSO) δ 6.30 (d, *J* = 15.5, 1H, HC=C), 7.03 (s, 1H, pyrrole), 7.14 (d, J = 13.5, 2H, Ar-H), 7.20-7.16 (m, *J* = 21, 4H), 7.41-7.24 (m, *J* = 83.5, 10H), 11.89 (s br, 1H, COOH); LCMS (ES$^+$) m/z = 400 $[M+H]^+$; HRMS: $C_{25}H_{17}NO_2Cl$ $[M-H]^+$ Calculated: 398.0953, Found: 398.0950.

2-((1-(4-Chlorophenyl)-2,5-diphenyl-1*H*-pyrrol-3-yl)methylene)malonamide (10c)

General Procedure E: **6a** (50 mg, 0.140 mmol), malonamide (18 mg, 0.175 mmol). Chromatography (EtOAc) and recrystallisation (MeOH) gave **10c** as white crystals (38 mg, 61%). mp 301.6 °C; IR (cm^{-1}) 3349, 3174, 2938, 2833, 1649, 1566; ^{1}H NMR (500MHz, d_6-DMSO) 6.90-6.79 (m, 2H, N'H' and 4-H), 7.11-7.04 (m, 6H, N'H and Ar-H), 7.16-7.13 (m, 2H, Ar-H), 7.29-7.20 (m, 3H, Ar-H), 7.31-7.30 (m, 5H, NH' and Ar-H), 7.58 (s, 1H, 3-CH), 7.97 (s, 1H, NH); ^{13}C NMR (125MHz, d_8-THF) 111.0, 119.1, 127.8, 128.7, 129.0, 129.7, 129.9, 131.3, 131.4, 131.9, 132.4, 133.6, 134.0, 136.7, 138.4, 140.1, 166.4, 172.1. LCMS (ES$^+$) m/z = 442.15 $[M+H^+]$; HRMS: $C_{26}H_{21}O_2N_3Cl$ [M+H] Calculated: 442.1317, Found: 442.1316

2-((1-(4-Chlorophenyl)-2,5-diphenyl-1H-pyrrol-3-yl)methylene)-*N*^{1},*N*^{3}-dimethylmalonamide (10d)

General Procedure E: **6a** (50 mg, 0.14 mmol), N,N'-dimethylmalondiamide (23mg, 0.175 mmol). Chromatography (5% MeOH, EtOAc) gave **10d** as a white solid (47 mg, 71%). mp 259.4 °C; IR (cm^{-1}) 3232, 3068, 2927, 1638, 1534. ^{1}H NMR (500MHz, $CDCl_3$) δ 2.84 (d, J = 5, 3H, NCH_3), 2.94 (d, J = 5, 3H, CH_3), 6.76-6.72 (m, 1H, NH), 6.54 (s, 1H, pyrrole), 6.84-6.82 (m, 2H, Ar-H), 7.07-7.02 (m, 4H, Ar-H), 7.16-7.12 (m, 2H, Ar-H), 7.22-7.19 (m, 3H, Ar-H), 7.28-7.25 (m, 3H, Ar-H), 7.35-7.30 (m, 1H, NH), 7.65 (s, 1H, 3-CH); ^{13}C NMR (125MHz, THF) δ 26.5, 30.8, 110.2, 119.1, 127.9, 128.8, 129.0, 129.1, 129.3, 129.7, 129.9, 131.1, 131.4, 131.9, 132.3, 133.5, 134.1, 136.8, 138.4, 140.2, 165.5, 170.2; LCMS m/z = 470.21 [M+H]; HRMS: $C_{28}H_{25}O_2N_3Cl$ [M+H] Calculated: 470.1630, Found: 470.1633.

2-((1-(4-Chlorophenyl)-2,5-diphenyl-1H-pyrrol-3-yl)methylene)-*N1,N3*-bis(2-hydroxyethyl)malonamide (10e)

General Procedure E: **6a** (50 mg, 0.140 mmol), *N*^{1},*N*^{3}-bis(2-hydroxyethyl)malonamide (33mg, 0.175mmol). Recrystallisation (EtOH, H_2O) gave **10e** as a yellow solid (36 mg, 49%). mp 246.3°C; IR (cm^{-1}) 3275; 2872 , 1637, 1598, 1525; ^{1}H NMR (500MHz, d_6-DMSO) δ 3.27-3.22 (m, 2H, CH_2), 3.41-3.37 (m, 2H, CH_2), 3.49-3.43 (m, 2H, CH_2), 3.63-3.56 (m, 2H, CH_2), 4.74 (t, 1H, J = 5 Hz, OH), 4.84 (t, 1H, J = 5 Hz, OH), 6.70 (s, 1H, pyrrole), 7.16-7.10 (m, 4H, Ar-H), 7.24-7.18 (m, 3H, Ar-H), 7.35-7.25 (m, 3H, Ar-H), 7.45-7.36 (m, 6H, Ar-H & 2NH), 8.51 (m, 1H, 3-CH). LCMS: m/z = 530.23 $[M+H^+]$ HRMS: $C_{30}H_{29}O_4N_3Cl$ [M+H] Calculated: 530.1841, Found: 530.1839.

This journal is © The Royal Society of Chemistry 2013

Ethyl 2-(cyclopropanecarbonyl)-4-oxo-4-phenylbutanoate (16a)

General Procedure F: ethyl 3-oxo-3-phenylpropanoate **15a** (4 g, 20.8 mmol), diethyl zinc (85 mL, 85 mmol), diiodomethane (6.7 mL, 85 mmol), cyclopropyl aldehyde (1.7 mL, 22.89 mmol), DCM (171 mL) and PCC (9.42 g, 40.3 mmol) gave **16a** as a clear viscous oil (2.04 g, 36%). IR (cm^{-1}) 1734, 1685, 1449, 1384; ^{1}H NMR (300 MHz) δ 0.94-1.20 (m, 4H, H_2 and H_2'), 1.34 (t, 7.0, CH_3), 2.25-2.41 (m, 1H, 2-(CO)CH), 3.5-3.78 (m, 2H, 3-H_2), 4.28 (q, 2H, H_2), 4.39-4.50, (m, 2H, 2-H), 7.40-7.65 (m, 3H, Ar-H), 7.92-8.80 (m, 2H, Ar-H); ^{13}C NMR (100 MHz) δ 11.8, 14.3, 20.9, 37.4, 54.9, 61.8, 128.4, 128.9, 133.5, 137.1, 169.4, 197.3, 204.2; LCMS (ES^+) m/z = 274.3 $[M+H]^+$

Ethyl 2-benzoyl-4-cyclopropyl-4-oxobutanoate (16b)

General Procedure F: ethyl 3-cyclopropyl-3-oxopropanoate **15b** (3.5 g, 22.5 mmol), diethyl zinc (92 mL, 92.1 mmol), diiodomethane (7.42 mL, 92.1 mmol), benzaldehyde (2.5 mL, 24.7 mmol), PCC (12.11 g, 56.2 mmol) gave **16b** as a clear viscous oil (2.93 g, 48%). ^{1}H NMR (400 MHz) δ 0.88-0.93 (m, 2H, H_2), 1.01-1.05 (m, 2H, H_2'), 1.14 (t, *J* = 7.1, 3H, CH_3), 1.99 (tt, *J* = 4.6 and 7.8, 1H, 4-CH), 3.28 (dd, *J* = 6.2 and 18.0, 1H, 3-H), 3.37 (dd, *J* = 7.5 and 18.0, 1H, 3-H'), 4.11 (q, *J* = 7.1, 2H, H_2), 4.91 (dd, *J* = 6.2 and 7.5, 1H, 2-H), 7.40-7.99 (m, 2H, Ar-H), 7.54-7.59 (m, 1H, Ar-H), 7.99-8.03 (m, 2H, Ar-H); ^{13}C NMR (100 MHz) δ 11.0, 13.9, 20.6, 41.9, 48.6, 61.6, 128.6, 128.8, 133.5, 136.1, 169.1, 194.7.

Methyl 2-benzoyl-5,5-dimethyl-4-oxohexanoate (16c)

General Procedure F: 4,4-dimethyl-3-oxopentanoate **15c** (1 g, 6.3 mmol), diethyl zinc (25.9 mL, 25.9 mmol), diiodomethane (2.08 mL, 25.9 mmol), benzaldehyde (0.67 mL, 6.8 mmol), PCC (3.25 g, 15.1 mmol) gave **16c** as a clear viscous oil (1.71 g, 98%). IR (cm^{-1}) 2958, 2872, 2019, 1738, 1684; ^{1}H NMR (300 MHz) δ 1.20 (s, 9H, *t*-Bu), 3.24 (dd *J* = 9, 18, 1H, 3-H), 3.35 (dd, *J* = 6, 18, 1H, 3-H'), 3.69 (s, 3H, CH_3), 4.94 (dd, *J* = 6, 9, 1H, 2-H), 7.48-7.54 (m, 2H, Ar-H), 7.59-7.65 (m, 1H, Ar-H), 8.00-8.80 (m, 2-H, Ar-H); ^{13}C (100 MHz) δ 26.8, 37.0, 44.3, 49.0, 52.8, 129.0, 129.2, 133.7, 136.7, 170.2, 195.1, 213.0

Ethyl 1-(4-chlorophenyl)-2-cyclopropyl-5-phenyl-1*H*-pyrrole-3-carboxylate (18d)

General Procedure G: **16a** (500 mg, 1.8 mmol), 4-chloroaniline (697 mg, 5.4 mmol), PTSA (17 mg, 0.09 mmol) in toluene (1.7 mL). Chromatography (4% EtOAc, hexane) gave **18d** (582 mg, 88%). ^{1}H NMR (400 MHz) δ 0.39-0.43 (m, 2H, H_2), 0.70-0.75 (m, 2H, H_2'), 1.37 (t, *J* = 7.1, 3H, CH_3), 1.79 (tt, *J* = 5.5 and 8.5, 1H, 2-CH), 4.32 (q, *J* = 7.1, 2H, H_2), 6.73 (s, 1H, 4-H), 7.00-7.04 (m, 2H, Ar-H), 7.07-7.10 (m, 2H, Ar-H), 7.13-7.19 (m, 3H, Ar-H), 7.29-7.34 (m, 2H, Ar-H); ^{13}C NMR (100 MHz) δ 7.9, 8.4, 14.5, 59.7, 110.7, 115.1, 126.8, 128.1, 128.4, 129.0, 129.6, 132.1, 133.6, 164.8.

Ethyl 1-(4-bromophenyl)-2-cyclopropyl-5-phenyl-1H-pyrrole-3-carboxylate (18e)

General Procedure G: **16a** (500 mg, 1.8 mmol), 4-bromoaniline (940 mg, 5.4 mmol), PTSA (17 mg, 0.09 mmol). Chromatography (4% EtOAc, hexane) gave **18e** (609 mg, 82%). IR (cm^{-1}) 3668, 2978, 2901, 1701, 1490, 1410; ^{1}H NMR (400 MHz) δ 0.39-0.44 (m, 2H, m, CH_2), 0.70-0.76 (m, 2H, H_2'), 1.36 (t, *J* = 7, 3H, CH_3) 1.79 (tt, *J* = 5.7, 8.4, 1H, 2-CH), 4.32 (q, *J* = 7, 2H, H_2), 6.72 (s, 1H, 4-H), 6.99-7.05 (m, 4H, Ar-H) 7.13-7.20 (m, 3H, Ar-H), 7.45-7.49 (m, 2H, Ar-H). ^{13}C NMR (75 MHz) δ 8.5, 8.7, 14.8, 60.0, 111.4, 115.7, 122.0, 127.2, 128.5, 130.0, 130.5, 132.4, 132.6, 134.0, 138.5, 141.2, 165.1. LCMS (ES^+) m/z = 412.2 $[M+H]^+$

Ethyl 1-(4-chlorophenyl)-5-cyclopropyl-2-phenyl-1H-pyrrole-3-carboxylate (18f)

General Procedure G: **16b** (500 mg, 1.8 mmol), 4-chloroaniline (697 mg, 5.4 mmol), PTSA (17 mg, 0.09 mmol). Chromatography (4% EtOAc, hexane) gave **18f** (582 mg, 88%). ^{1}H NMR (400 MHz) δ 0.79-0.84 (m, 2H, H_2) 0.85-0.91 (m, 2H, H_2'), 1.30 (t, *J* = 7.2, 3H, CH_3), 1.54-1.62 (m, 1H, 5-CH), 4.28 (q, *J* = 7.2, 2H, H_2), 6.53 (s, 1H, 4-H), 7.19-7.23 (m, 2H, H_2), 7.27-7.31 (m, 2H, Ar-H), 7.27-7.31 (m, 2H, Ar-H), 7.31-7.35 (m, 3H, Ar-H), 7.38-7.42 (m, 2H, Ar-H); ^{13}C NMR (75 MHz) δ 7.6, 8.2, 14.4, 59.7, 106.8, 114.0, 127.37, 127.9, 129.2, 130.3, 131.5, 132.2, 134.1, 137.3, 137.5, 138.9, 165.0.

Methyl 1-(4-bromophenyl)-5-cyclopropyl-2-phenyl-1*H*-pyrrole-3-carboxylate (18g)

General Procedure g: **16b** (500 mg, 1.8 mmol), 4-bromoaniline (941 mg, 5.4 mmol), PTSA (17 mg, 0.09 mmol). Chromatography (4% EtOAc, hexane) gave **18g** (582 mg, 88%). ^{1}H NMR (400 MHz) δ 0.63-0.68 (m, 2H, CH_2), 0.69-0.75 (m, 2H, H_2), 1.14 (t, *J* = 7.2, 1H, CH_3), 1.38-1.46 (m, 2H, 5-CH), 4.12 (q, *J* = 7.2, 2H, CH_2), 6.37 (s, 1H, 4-H), 6.97-7.01 (m, 2H, Ar-H), 7.11-7.16 (m, 2H, Ar-H), 7.17-7.21 (m, 3H, Ar-H), 7.38-7.42 (m, 2H, Ar-H). ^{13}C NMR (400 MHz) δ 7.4, 7.9, 14.2, 59.5, 106.3, 113.2, 121.6, 127.4, 127.7, 130.2, 131.1, 131.4, 131.9, 137.0, 137.2, 138.6, 164.7.

(1-(4-Bromophenyl)-5-(*tert*-butyl)-2-phenyl-1*H*-pyrrol-3-yl)methanol (19b)

General Procedure H: **18b** (100 mg, 0.24 mmol), DIBAL-H (0.61 mL, 0.61 mmol). Chromatography (30% EtOAc, hexane) gave **19b** (75 mg, 80%). IR (cm^{-1}) 3430, 2961, 2920, 2866, 1485; ^{1}H NMR (300 MHz) δ 1.10 (s, 9H, *t*-Bu), 1.51 (br. s, 1H, OH) 4.40 (s, 2H, 3-CH_2), 6.18 (s, 1H, 4-H), 6.98-7.12 (m, 7H, Ar-H), 7.28-7.35 (m, 2H, Ar-H); ^{13}C NMR (100 MHz) δ 31.8, 33.2, 58.6, 106.7, 121.0, 122.3, 127.3, 128.1, 131.3, 131.6, 132.3, 132.9, 134.9, 140.4, 144.4.

(5-(*tert*-Butyl)-1-(4-chlorophenyl)-2-phenyl-1*H*-pyrrol-3-yl)methanol (19c)

General Procedure H: **18c** (50 mg, 0.14 mmol), DIBAL-H (0.34 mL, 0.34 mmol). Chromatography (30% EtOAc, hexane) gave **19c** (42 mg, 91%). IR (cm^{-1}) 2957, 2925, 1491; ^{1}H NMR (300 MHz) δ 1.10 (s, 9H, *t*-Bu), 1.46 (br. s, 1H, OH), 4.39 (s, 2H, 3-CH2), 6.18 (s, 1H, 4-H), 6.93-7.01 (m, 2H, Ar-H), 7.05-7.18 (m, 7H, Ar-H); ^{13}C (100 MHz) δ 31.8, 33.2, 58.6, 106.6, 127.3, 128.0, 128.5, 131.3, 132.6, 134.3, 135.0, 144.4

(1-(4-Chlorophenyl)-2-cyclopropyl-5-phenyl-1*H*-pyrrol-3-yl)methanol (19d)

General Procedure H: **18d** (300 mg, 0.71 mmol), DIBAL-H (2.2 mL, 2.2 mmol). Chromatography (30% EtOAc, hexane) gave **19d** (250 mg, 91%). ^{1}H NMR (400 MHz) δ 0.44-0.50 (m, 2H, H_2), 0.62-0.68 (m, 2H, H_2'), 1.51 (br. s, 1H, OH), 1.58-1.67 (m, 1H, 2-CH), 4.69, (s, 2H, 3-CH_2), 6.36 (s, 1H, 4-H), 7.01-7.05 (m, 2H, Ar-H), 7.08-7.19 (m, 5, Ar-H), 7.28-7.33 (m, 2H, Ar-H); ^{13}C NMR (100 MHz) 6.3, 6.6, 38.3, 57.7, 109.7, 122.41, 126.31, 128.3, 129.0, 129.8, 132.8, 133.0, 133.9.

(1-(4-Bromophenyl)-2-cyclopropyl-5-phenyl-1H-pyrrol-3-yl)methanol (19e)

General Procedure H: **18e** (304 mg, 0.74 mmol), DIBAL-H (2.2 mL, 2.2 mmol). Chromatography (30% EtOAc, hexane) gave **19e** (250 mg, 91%). ^{1}H NMR (400 MHz) δ 0.44-0.49 (m, 2H, H_2), 0.63-0.68 (m, 2H, H_2'), 1.48 (br. s, 1H, OH), 1.59-1.66 (m, 1H, 2-CH), 4.69 (s, 2H, 3-CH_2), 6.36 (s, 1H, 4-H), 7.00-7.06 (m, 4H, Ar-H), 7.09-7.19 (m, 3H, Ar-H), 7.43-7.48 (m, 2H, Ar-H); ^{13}C NMR (100 MHz) δ 6.4, 6.6, 57.7, 109.7, 120.9, 122.3, 126.2, 128.1, 130.0, 131.8, 132.7, 133.4, 133.7, 138.7.

(1-(4-Chlorophenyl)-5-cyclopropyl-2-phenyl-1H-pyrrol-3-yl)methanol (19f)

General Procedure H: **18f** (291 mg, 0.79 mmol), DIBAL-H (2.4 mL, 2.39 mmol). Chromatography (30% EtOAc, hexane) gave **19f** (233 mg, 90%). ^{1}H NMR (300 MHz) δ 0.52-0.61 (m, 2H, H_2), 0.63-0.70 (m, 2H, H_2'), 1.38-1.49 (m, 1H, 5-CH), 1.58 (br s. 1H, OH), 4.43 (s, 2H, 3-CH_2), 5.97 (s, 1H, 4-H), 6.98-7.08 (m, 5H, Ar-H), 7.10-7.14 (m, 2H, Ar-H), 7.16-7.23 (m, 2H, Ar-H); ^{13}C (100 MHz) δ 7.7, 8.5, 58.5, 105.8, 122.1, 127.1, 128.1, 128.3, 129.1, 129.1, 130.2, 130.6, 132.5, 132.6, 133.1, 133.2, 133.4, 137.7, 138.3.

(1-(4-Bromophenyl)-5-cyclopropyl-2-phenyl-1*H*-pyrrol-3-yl)methanol (19g)

General Procedure H: **18g** (300 mg, 0.73 mmol), DIBAL-H (2.2 mL, 2.2 mmol). Chromatography (30% EtOAc, hexane) gave **19g** (229 mg, 85%). ^{1}H NMR (300 MHz) δ 0.65-0.70 (m, 2H, H_2), 0.71-0.80 (m, 2H, H_2'), 1.44-1.59 (m, 2H, 5-H), 1.64 (br. s, 1H, OH), 4.53 (s, 2H, 3-CH_2), 6.07 (s, 1H, 4-H), 7.03-7.12 (m, 4H, Ar-H), 7.19-7.30 (m, 3H, Ar-H), 7.40-7.47 (m, 2H, Ar-H); ^{13}C NMR (75 MHz) δ 7.8, 8.5, 58.5, 105.9, 121.3, 122.2, 127.1, 128.3, 130.5, 130.6, 132.2, 132.2, 132.6, 137.7, 138.8;

1-(4-Bromophenyl)-5-(*tert*-butyl)-2-phenyl-1*H*-pyrrole-3-carbaldehyde (17b)

General Procedure I: **19b** (75 mg, 0.20 mmol), TPAP (7 mg, 0.19 mmol), NMO (46 mg, 0.4 mmol) and 4 Å molecular sieves (100 mg). Chromatography (5-10% EtOAc, hexane) gave **17b** (62mg, 83%). ^{1}H NMR (300 MHz) 1.09 (s, 9H, *t*-Bu), 6.58 (s, 1H, 4-H), 7.00-7.08 (m, 4H, Ar-H), 7.11-7.17 (m, 3H, Ar-H), 7.31-7.39 (m, 2H, Ar-H), 9.43 (s, 1H, CHO); ^{13}C NMR (100 MHz) 31.4, 33.3, 104.5, 123.2, 123.6, 128.2, 128.8, 131.7, 131.9, 132.6, 138.7, 146.3, 186.9.

5-(*tert*-Butyl)-1-(4-chlorophenyl)-2-phenyl-1H-pyrrole-3-carbaldehyde (17c)

General Procedure I. **19c** (42 mg, 0.12 mmol), TPAP (4 mg, 0.12 mmol), NMO (28 mg, 0.25 mmol) and 4 Å molecular sieves (61 mg). Chromatography (5-10% EtOAc, hexane) gave **17c** (21 mg, 50%). IR (cm^{-1}) 698, 746, 1090, 1217, 1431, 1486, 1661, 2928, 2963; ^{1}H NMR (300 MHz) δ 1.10 (s, 9H, *t*-Bu), 6.59 (s, 1H, 4-H), 7.04-7.21 (m, 9H, Ar-H), 9.44 (s, 1H, CHO); ^{13}C NMR (100 MHz) δ 31.4, 33.3, 104.5, 123.7, 128.2, 128.7, 128.9, 130.0, 131.7, 132.2, 135.2, 138.2, 145.8, 146.3, 186.9; LCMS (ES^{+}) m/z = 337.84 $[M+H]^{+}$;

1-(4-Bromophenyl)-2-cyclopropyl-5-phenyl-1H-pyrrole-3-carbaldehyde (17e)

General Procedure I: **19e** (196 mg, 0.53 mmol), TPAP (18 mg, 0.53 mmol), NMO (114 mg; 1.06 mmol) and 4A MS (266 mg). Chromatography (10-20% EtOAc, hexane) gave **17e** (168 mg, 86%). ^{1}H NMR (400 MHz) δ 0.54-0.59 (m, 2H, H_2), 0.78-0.84 (m, 2H, $H_2{'}$), 1.77-1.84 (m, 1H, 2-CH), 6.74 (s, 1H, 4-H), 6.99-7.07 (m, 4H, Ar-H), 7.15-7.21 (m, 3H, Ar-H), 7.48-7.53 (m, 2H, Ar-H), 10.2 (s, 1H, CHO); ^{13}C NMR (100 MHz) δ 6.8, 7.3, 53.4, 107.4, 122.1, 124.3, 127.2, 128.3, 128.4, 129.7, 131.5, 132.2, 135.5, 137.4, 145.0, 186.2;

1-(4-Chlorophenyl)-2-cyclopropyl-5-phenyl-1*H*-pyrrole-3-carbaldehyde (17d)

General Procedure I: **19d** (176 mg, 0.48 mmol), TPAP (17 mg, 0.47 mmol), NMO (112 mg; 0.95 mmol). Chromatography (10-20% EtOAc, hexane) gave **17d** (146 mg; 98%). ^{1}H NMR (400MHz) δ 0.54-0.59 (m, 2H, H_2), 0.77-0.84 (m, 2H, H_2'), 1.77-1.84 (m, 1H, 2-CH), 6.74 (s, 1H, 4-H), 7.00-7.05 (m, 2H, Ar-H), 7.09-7.13 (m, 2H, Ar-H), 7.15-7.20 (m, 3H, Ar-H), 7.32-7.37 (m, 2H, Ar-H), 1.20 (m, 1H, CHO), ^{13}C NMR (100 MHz) δ 6.8, 7.3, 29.7, 107.4, 124.4, 127.3, 128.3, 128.6, 129.3, 129.6, 131.6, 134.1, 135.7, 137.0, 144.7, 186.9

1-(4-Chlorophenyl)-5-cyclopropyl-2-phenyl-1H-pyrrole-3-carbaldehyde (17f)

General Procedure I: **19f** (176 mg, 0.48 mmol), TPAP (17 mg, 0.47 mmol), NMO (112 mg, 0.95 mmol) and 4A MS (238 mg) in DCM (1.1 mL). Chromatography (10-20% EtOAc, hexane) gave **17f** (146 mg, 95%). ^{1}H NMR (300 MHz) δ 0.59-0.65 (m, 2H, H2), 0.67-0.74 (m, 2H, H2), 1.32-1.42 (m, 2H, 5-CH), 6.36 (s, 1H, 4-H), 7.01-7.11 (m, 4H, Ar-H), 7.15-7.28 (m, 5H, Ar-H), 9.54 (s, 1H, CHO); ^{13}C (100 MHz) δ 7.8, 8.2, 30.0, 103.4, 124.2, 128.5, 128.8, 129.6, 129.9, 130.2, 131.3, 134.6, 136.7, 139.7, 143.5, 186.7.

1-(4-Bromophenyl)-5-cyclopropyl-2-phenyl-1*H*-pyrrole-3-carbaldehyde (17g)

General Procedure I. **19g** (200 mg, 0.54 mmol), TPAP (19 mg, 0.054 mmol), NMO (127 mg, 1.08 mmol) and 4 Å molecular sieves (271 mg). Chromatography (5-10% EtOAc, hexane) gave **17g** (152 mg, 76%). ^{1}H NMR (300 MHz) δ 0.57-0.65 (m, 2H, H_2), 1.32-1.43 (m, 1H, 5-CH), 6.36 (s, 1H, 4-H), 6.97-7.02 (m, 2H, Ar-H), 7.03-7.12 (m, 2H, Ar-H), 7.18-7.25 (m, 3H, Ar-H), 7.37-7.42 (m, 2H, Ar-H), 9.55 (s, 1H, CHO); ^{13}C NMR (75 MHz) δ 7.9, 8.2, 103.4, 122.5, 124.2, 128.5, 128.8, 129.9, 130.5, 131.3, 132.5, 137.2, 139.7, 143.4, 186.7.

5-((1-(4-Bromophenyl)-5-(*tert*-butyl)-2-phenyl-1*H*-pyrrol-3-yl)methylene)-2-thioxodihydropyrimidine-4,6(1*H*,5*H*)-dione (11b)

General Procedure J: **17b** (67 mg, 0.20 mmol) and thiobarbituric acid (31 mg, 0.22 mmol) to give **24b** as bright yellow solid (46 mg, 50%).LCMS 508.43; 309.0 – 309.8 °C; IR (cm^{-1}) 3155, 3064, 2967, 2869, 1694, 1645, 1516, 1484; ^{1}H NMR (300 MHz, THF d^8) δ 11.28 (1H, br s, N*H*), 11.22 (1H, br s, N*H*), 8.11 (1H, s, 3-C*H*), 7.99 (1H, s, 4-*H*), 7.49-7.51 (2H, m, Ar-*H*) 7.25-7.31 (5H, m, Ar-*H*), 7.13-7.16 (2H, m, Ar-*H*), 1.22 (9H, s, 5-(CH_3)$_3$); LCMS (ES^+) m/z = 508.43 $[M+H]^+$; HRMS: $C_{25}H_{22}{}^{79}BrN_3O_2S$ $[M+H]^+$ Calculated: 508.4301, Found: 508.0869.

5-((5-(*tert*-Butyl)-1-(4-chlorophenyl)-2-phenyl-1*H*-pyrrol-3-yl)methylene)-2-thioxodihydropyrimidine-4,6(1*H*,5*H*)-dione (11c)

General Procedure I: **17c** (21 mg, 0.06 mmol) and thiobarbituric acid (10 mg, 0.067 mmol) gave **11c** as bright yellow solid (13 mg, 47%). mp: 308.9-309.2 °C; IR (cm^{-1}) 3064, 2968, 2870, 1694, 1642, 1516, 1487; ^{1}H NMR(300 MHz, THF d^8) δ 11.28 (1H, br s, NH), 11.21 (1H, br s, NH), 8.11(1H, s, 3-CH), 7.99 (1H, s, 4-H), 7.35(4H, s, Ar-H) 7.25-7.35 (3H, m, Ar-H), 7.14-7.16 (2H, m, Ar-H), 1.22 (9H, s, 5-(CH_3)$_3$); HRMS: $C_{25}H_{23}{}^{35}ClN_3O_2S$ $[M+H]^+$ Calculated: 464.1194, Found: 464.1195.

5-((1-(4-Chlorophenyl)-2-cyclopropyl-5-phenyl-1*H*-pyrrol-3-yl)methylene)-2-thioxodihydropyrimidine-4,6(1*H*,5*H*)-dione (11d)

General Procedure J. **17d** (115 mg, 0.357 mmol), thiobarbituric acid (56 mg, 0.393 mmol) to give **11d** a bright orange solid (119 mg, 74%). mp 298 ^{o}C; IR (cm^{-1}) 3134, 1696, 1636, 1516, 1487, 1441. ^{1}H NMR (400 MHz, THF d^8) δ 0.48-0.52 (2H, m, H_2), 0.86-0.91 (2H, m, H_2'), 1.95 (1H, tt, *J* = 8.4, 5.5, 2-CH), 7.10-7.11 (2H, m, Ar-H), 7.16-7.22 (3H, m, Ar-H), 7.27-7.31 (2H, m, Ar-H), 7.42-7.46 (2H, m, Ar-H), 8.04 (1H, s, 4-H), 9.04 (1H, s, 3-CH), 11.27 (1H, br s, NH), 11.38 (1H, br s, NH); LCMS (ES^+) m/z = 447.9 $[M+H]^+$; HRMS**:** $C_{24}H_{19}ClN_3O_2S$ $[M+H]^+$ Calculated: 448.0881, Found**:** 448.0881.

5-((1-(4-Bromophenyl)-2-cyclopropyl-5-phenyl-1*H*-pyrrol-3-yl)methylene)-2-thioxodihydropyrimidine-4,6(1*H*,5*H*)-dione (11e)

General Procedure J: **17e** (111 mg, 0.30 mmol) and thiobarbituric acid (48 mg, 0.33 mmol) to give **11e** as a bright orange solid (103 mg, 70%). mp 304-305 °C; IR (cm^{-1}) 3133, 3059, 2892, 1665, 1635, 1510, 1485; ^{1}H NMR (400MHz, THF d^8) δ 0.48-0.52, (2H, m, H_2) 0.86-0.91 (2H, m, H_2'), 1.95 (1H, tt, *J* = 8.4, 5.6, 2-CH), 7.09-7.11 (2H, m, Ar-H), 7.16-7.20 (3H, m, Ar-H), 7.21-7.24 (2H, m, Ar-H), 7.53- 7.60 (2H, m, Ar-H), 8.03 (1H, s, 4-H), 9.03(1H, s, 3-CH), 11.27 (1H, br s, NH), 11.38 (1H, br s, NH); LCMS (ES^+) m/z = 494.2 $[M+H]^+$; HRMS: $C_{24}H_{18}BrN_3O_2S$ $[M+H]^+$ Calculated: 492.0376, Found: 492.0367

5-((1-(4-Chlorophenyl)-5-cyclopropyl-2-phenyl-1*H*-pyrrol-3-yl)methylene)-2-thioxodihydropyrimidine-4,6(1*H*,5*H*)-dione (11f)

General Procedure J: **17f** (115 mg, 0.36 mmol), thiobarbituric acid (56 mg, 0.39 mmol) gave **11f** as a bright orange solid (119 mg, 74%). mp 300 °C; IR (cm^{-1}) 3671, 2984, 2901, 1692, 1636, 1518, 1485, 1439; ^{1}H NMR (300 MHz, THF d^{8}) 0.68-0.79 (4H, m, H_2 and H_2'), 1.47-1.56 (1H, m, 5-CH), 7.15-7.19 (2H, m, Ar-H), 7.26-7.35 (5H, m, Ar-H), 7.37-7.42 (2H, s, Ar-H), 7.73 (1H, s, 4-H), 8.21(1H, s, 3-CH), 11.21 (1H, br s, NH), 11.28 (1H, br s, NH); LCMS (ES^{+}) m/z = 448.27 [M+H]$^{+}$; HRMS: $C_{24}H_{18}{}^{35}ClN_3O_2S$ [M+H]$^{+}$ Calculated: 448.0881, Found: 448.0880.

5-((1-(4-Bromophenyl)-5-cyclopropyl-2-phenyl-1*H*-pyrrol-3-yl)methylene)-2-thioxodihydropyrimidine-4,6(1*H*,5*H*)-dione (11g)

General Procedure J: **17g** (200 mg, 0.54 mmol), thiobarbituric acid (78 mg, 0.54 mmol). Chromatography (10-20% EtOAc, hexane) gave **11g** (152 mg, 76%). mp 312 °C; IR (cm^{-1}) 3662, 2984, 2901, 1692, 1636, 1516, 1481; ^{1}H NMR (300 MHz, THF d^{8}) δ 0.68-0.79 (4H, m, H_2 and H_2'), 1.47-1.57 (1H, m, 5-CH), 7.14-7.17 (2H, m, Ar-H), 7.19-7.24 (2H, m, Ar-H), 7.29-7.34 (3H, m, Ar-H), 7.52-7.56(2H, m, Ar-H), 7.73 (1H, s, 4-H), 8.20 (1H, s, 3-CH), 11.21 (1H, br s, NH), 11.27 (1H, br s, NH); LCMS (ES^{+}) m/z = 492.20 [M+H]$^{+}$; HRMS: $C_{24}H_{18}{}^{79}BrN_3O_2S$ [M+H]$^{+}$ Calculated: 492.0376, Found: 492.0368.

Combustion Analysis Data

Paper #	Mol Form.	Requires			Found		
		C	H	N	C	H	N
**4w**	$C_{31}H_{26}BrN_3O_2S$	63.70	4.48	7.19	64.02	4.53	7.10
**4y**	$C_{28}H_{20}BrN_3O_3$	63.89	3.83	7.98	63.65	3.95	7.61
**4z**	$C_{28}H_{20}BrN_3O_2S$	62.01	3.72	7.75	62.31	3.70	7.87

Table S1: MDM2 structure-activity relationships for commercial pyrroles **4a-l**.

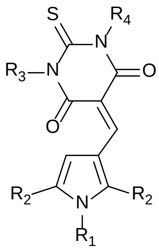

**Compound**	**R^1**	**R^2**	**R^3**	**R^4**	**MDM2 IC$_{50}$ (nM)**
**4a**	4-ClPh	Me	H	H	720 ± 100
**4b**	4-ClPh	Me	H	Ph	3300 ± 700
**4c**	4-ClPh	Ph	H	H	120 ± 20
**4d**	4-ClPh	Ph	H	Ph	230 ± 44
**4e**	4-ClPh	Ph	H	3-ClPh	163 ± 17
**4f**	4-ClPh	Ph	3,4-diMePh	Ph	256 ± 39
**4g**	4-ClPh	Ph	Me	Me	Insol.
**4h**	4-BrPh	Me	Ph	Ph	199 ± 16
**4i**	4-MePh	Me	H	Ph	8400 ± 900
**4j**	4-MePh	Me	H	H	4700 ± 200
**4k**	4-EtO$_2$CPh	Ph	H	H	700 ± 20

Table S2: Structures of additional commercial analogues with MDM2-p53 IC_{50} values

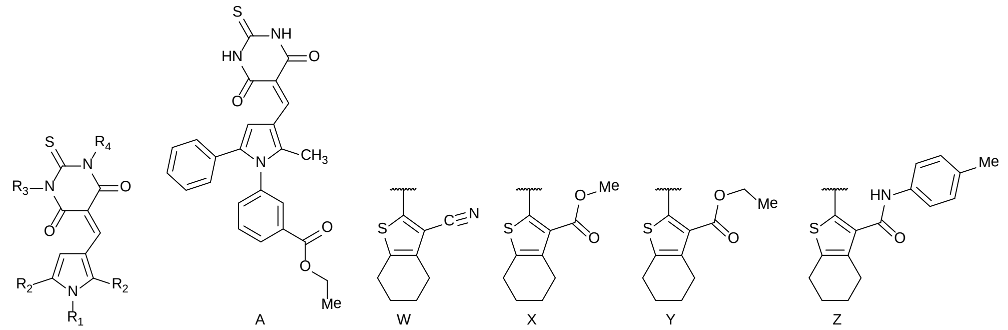

A W X Y Z

R^1	R^2	R^3	R^4	IC_{50} (μM)
4-BrPh	Me	H	H	>10
4-BrPh	Me	Ph	Ph	0.20 ± 0.02
Ph	Me	H	H	>10
3,5-diMePh	Me	H	H	>10
3,5-diMePh	Me	Ph	Ph	>10
3,5-diMePh	Me	H	Ph	>10
3,5-diMePh	Me	H	4-BrPh	>10
3,5-diMePh	Me	H	3-ClPh	>10
3,5-diMePh	Me	H	2-FPh	>10
4-AcOPh	Me	H	H	>10
4-AcOPh	Me	Ph	Ph	>10
4-AcOPh	Me	H	Ph	>10
4-AcOPh	Me	H	4-BrPh	>10
3-NO_2Ph	Me	H	H	>10
3-NO_2Ph	Me	Ph	Ph	>10
3-NO_2Ph	Me	H	Ph	>10
3-NO_2Ph	Me	H	4-BrPh	>10
3-NO_2Ph	Me	H	3-ClPh	>10
4-EtO_2CPh	Me	H	H	>10
4-EtO_2CPh	Me	Ph	Ph	>10
4-EtO_2CPh	Me	H	Ph	>10
4-EtO_2CPh	Me	H	4-BrPh	>10
4-EtO_2CPh	Me	H	3-EtOPh	>10
4-EtO_2CPh	Me	Me	Me	>10
4-ClPh	Ph	H	H	0.12 ± 0.02
4-ClPh	Ph	H	Ph	0.23 ± 0.04
4-ClPh	Ph	H	4-BrPh	>10
4-ClPh	Ph	H	3-ClPh	0.16 ± 0.02
4-ClPh	Ph	3,4-diMePh	Ph	0.26 ± 0.04
4-ClPh	Ph	Me	Me	Insol.
4-EtO_2CPh	Ph	H	H	0.70 ± 0.02
4-EtO_2CPh	Ph	H	3-EtOPh	>10
4-EtO_2CPh	Ph	H	4-BrPh	>10
4-EtO_2CPh	Ph	H	3MeOPh	>10
4-MePh	Me	H	3-EtOPh	>10
4-MePh	Me	H	4-FPh	>10
4-MePh	Me	H	H	4.7 ± 0.2

R^1	R^2	R^3	R^4	IC_{50} (μM)
4-MePh	Me	H	Ph	8.4 ± 0.9
4-ClPh	Me	H	H	0.72 ± 0.10
4-ClPh	Me	H	Ph	3.3 ± 0.7
4-ClPh	Me	Me	Me	>10
3,4-diMePh	Me	H	3-EtOPh	>10
4-Me_2NPh	Me	H	3-EtOPh	>10
4-Me_2NPh	Me	H	4-BrPh	>10
4-(4-NO_2PhS) Ph	Me	H	4-MePh	>10
C_6H_{11}	Me	H	H	>10
C_6H_{11}	Me	H	4-BrPh	>10
C_6H_{11}	Me	H	4-EtPh	>10
C_6H_{11}	Me	H	Ph	>10
4-BrPh	Me	H	4-EtOPh	>10
4-HO_2CPh	Me	H	4-EtPh	>10
3-HO_2CPh	Me	H	4-EtPh	>10
3-Me-4-IPh	Me	H	4-MeOPh	>10
4-*t*-BuPh	Me	H	2,3-diClPh	>10
4-MeO_2CPh	Me	H	H	>10
3,5-di(HO_2C)Ph	Me	H	2-FPh	>10
2-Et-5-MePh	Me	H	H	>10
2-Et-5-MePh	Me	H	4-MeOPh	>10
2-Et-5-MePh	Me	H	2-F-Ph	>10
2-F-Ph	Me	H	2-F-Ph	>10
4-F-Ph	Me	H	2-F-Ph	>10
3-(HO_2C)-4-ClPh	Me	H	2-F-Ph	>10
2-Me-3-(HO_2C) Ph	Me	H	2-F-Ph	>10
4-EtPh	Me	H	4-F-Ph	>10
Ph	Me	Me	Me	>10
4-NO_2Ph	Me	Me	Me	>10
4-MePh	Me	Me	Me	>10
3-NO_2Ph	Me	Me	Me	>10
2-MePh	Me	Me	Me	>10
3-HO_2CPh	Me	Me	Me	>10
4-HO_2CPh	Me	H	Me	>10
4-HO_2CPh	Me	H	Et	>10
4-HOPh	Me	Me	Me	>10
2,6-diMePh	Me	Me	Me	>10
4-Ph-Ph	Me	H	H	>10
2-Et-Ph	Me	H	H	>10
3-AcOPh	Me	H	H	>10
Me	Me	3-MeOPh	H	>10
3,5-(HO_2C)$_2$Ph	Me	H	H	>10
4-(4-NO_2PhS)-Ph	Me	2-ClPh	H	>10
4-(4-BrBn)-OPh	Me	H	3-MePh	>10
4-Py	Me	Me	Me	>10
3-Py	Me	Me	Me	>10
3-Py	Me	H	$CH_2CH=CH_2$	>10
3-Py	Me	H	3-FPh	>10
2-Py	Me	Me	Me	>10
2-Py	Me	H	$CH_2CH=CH_2$	>10

R^1	R^2	R^3	R^4	IC_{50} (μM)
$MeOCH_2CH_2$	Me	Me	Me	>10
$MeOCH_2CH_2$	Me	H	$CH_2CH=CH_2$	>10
3,4-(OCH_2O)Ph	Me	H	4-BrPh	>10
W	Me	H	4-EtPh	>10
W	Me	Ph	Ph	>10
W	Me	H	4-ClPh	>10
X	Me	H	Me	>10
Y	Me	H	Ph	>10
Z	Me	H	H	>10
Z	Me	H	3,5-diMePh	>10
Z	Me	H	4-MeOPh	>10
Z	Me	H	4-BrPh	>10
A	-	-	-	>10

Table S3: Growth inhibitory activity of selected purchased pyrroles in a panel of cell lines with defined MDM2 and p53 status.

Compound	GI_{50}				
	**SJSA-1**[a]	**A2708**	**A2780-CP70**[b]	**HCT116**	**HCT116 p53(-/-)**[b]
**3**	4.4 ± 0.1	3.9 ± 0.1	3.9 ± 0.1	7.8 ± 0.2	7.7 ± 0.1
**4a**	3.3 ± 0.1	3.4 ± 0.2	4.4 ± 0.2	8.4 ± 0.5	8.3 ± 0.4
**4b**	7.6 ± 0.1	8.4 ± 0.2	9.5 ± 0.1	9.5 ± 0.3	9.9 ± 0.1
**4i**	7.1 ± 0.1	7.5 ± 0.1	9.0 ± 0.4	9.5 ± 0.3	9.7 ± 0.2

b) p53(-/-)

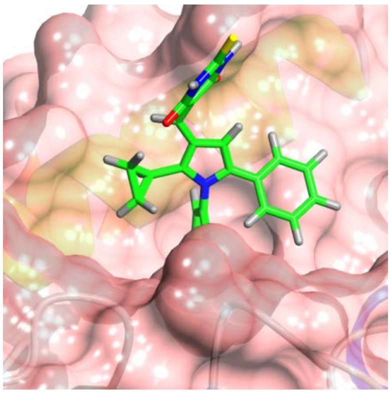

Figure 1: Modeled binding mode of **11d** (green) overlayed with MDMX (pink).

This journal is © The Royal Society of Chemistry 2013

REFERENCES

1. I. R. Hardcastle, S. U. Ahmed, H. Atkins, G. Farnie, B. T. Golding, R. J. Griffin, S. Guyenne, C. Hutton, P. Kallblad, S. J. Kemp, M. S. Kitching, D. R. Newell, S. Norbedo, J. S. Northen, R. J. Reid, K. Saravanan, H. M. G. Willems and J. Lunec, *J. Med. Chem.*, 2006, **49**, 6209-6221.
2. P. Emsley, B. Lohkamp, W. G. Scott and K. Cowtan, *Acta Crystallographica Section D*, 2010, **66**, 486-501.
3. M. D. Winn, C. C. Ballard, K. D. Cowtan, E. J. Dodson, P. Emsley, P. R. Evans, R. M. Keegan, E. B. Krissinel, A. G. W. Leslie, A. McCoy, S. J. McNicholas, G. N. Murshudov, N. S. Pannu, E. A. Potterton, H. R. Powell, R. J. Read, A. Vagin and K. S. Wilson, *Acta Crystallographica Section D*, 2011, **67**, 235-242.
